# GWAIS-Web: a free and secure web service for ultra-fast and large-scale genome-wide association interaction studies

**DOI:** 10.1093/bioadv/vbag172

**Published:** 2026-06-17

**Authors:** Lars Wienbrandt, Christoph Prieß, David Ellinghaus

**Affiliations:** Institute of Clinical Molecular Biology, Kiel University and University Medical Center Schleswig-Holstein, Rosalind-Franklin-Str. 12, Kiel, 24105, Germany; Institute of Clinical Molecular Biology, Kiel University and University Medical Center Schleswig-Holstein, Rosalind-Franklin-Str. 12, Kiel, 24105, Germany; Institute of Clinical Molecular Biology, Kiel University and University Medical Center Schleswig-Holstein, Rosalind-Franklin-Str. 12, Kiel, 24105, Germany

## Abstract

**Summary:**

Genome-wide association interaction studies (GWAIS) are essential for identifying non-additive genetic effects underlying complex diseases, but exhaustive screening of SNP-SNP combinations across millions of variants is computationally demanding. We present GWAIS-Web, a free, ultra-fast, privacy-compliant web service that enables genome-wide screening of pairwise or third-order interactions for large case-control GWAS datasets. GWAIS-Web combines algorithmic advances with GPU- and FPGA-accelerated implementations, achieving speedups of more than three orders of magnitude compared to established CPU-based approaches and reducing analysis runtimes from years to days. The service supports multiple widely used epistasis detection methods, including PLINK’s logistic regression test and BOOST, and provides practical options such as LD-based SNP filtering, region-specific analyses and the simultaneous execution of multiple methods within a single run.

**Availability and implementation:**

GWAIS-Web is freely available at https://hybridcomputing.ikmb.uni-kiel.de. Source code for the stand-alone CPU/GPU implementation and the FPGA extension is provided at https://github.com/ikmb/hybridgwais and https://github.com/ikmb/hybridgwais-fpga.

## 1 Introduction

Additive genetic effects of Single Nucleotide Polymorphisms (SNPs) identified in Genome-Wide Association Studies (GWAS) explain only a fraction of the estimated heritability, which is referred to as missing heritability (Tam *et al*. 2019). Statistical interaction refers to the interaction variance explained by combinations of variants at different genetic loci (non-additive effects) rather than their single marker effects. For example, in psoriasis a significant interaction detected by logistic regression has been reported between the genes *ERAP1* (rs27524) and *HLA-C* (rs10484554) with genotypic odds ratios (ORs) of up to 18.0 relative to the most protective two-locus genotype (Genetic Analysis of Psoriasis Consortium *et al*. 2010). The biological implication of this interaction is that the *ERAP1* risk allele of SNP rs27524 only has a strong effect in individuals carrying at least one copy of the risk allele of SNP rs10484554 at the *HLA-C* locus. Although numerous studies have identified prognostic and immunological associations of individual molecular markers with human diseases (e.g. lung cancer (Wu *et al*. 2020)), comprehensive investigations of interactions between genetic factors—particularly SNP–SNP interactions or epistatic effects—remain comparatively scarce. Epistasis is common in model organisms such as yeast, fruit flies, and mice (Mackay 2014), but the systematic identification of statistical interactions in human GWAS data has been difficult. One major challenge is the substantial computational burden associated with Genome-Wide Association Interaction Studies (GWAIS), which require testing an enormous number of SNP combinations and therefore typically demand very large cohorts, often on the scale of the UK Biobank with up to 500 000 GWAS samples (Backman *et al*. 2021). In their evaluations of epistasis detection methods, Wei *et al*. (2014) and Ponte-Fernández *et al*. (2022) further showed that exhaustive search strategies generally tend to provide the most reliable results, despite their particularly high computational cost.

We previously combined algorithmic improvements of the commonly used multiplicative logistic regression SNPxSNP interaction test in PLINK (Chang *et al*. 2015) with hardware acceleration and achieved a fundamental speedup (Wienbrandt *et al*. 2019). Among other things, we replaced the previously used covariate matrices in PLINK 1.9 with contingency tables and implemented the generation of these contingency tables on either a Field-Programmable Gate Array (FPGA) or a Graphics Processing Unit (GPU) with the computation of the test statistic also on a GPU. One limitation, however, is that many users have not yet been able to benefit from the accelerated implementation, because they do not have access to these special high-performance computing resources or cannot implement the algorithms we propose on FPGAs and/or GPUs themselves.

To enable large-scale GWAIS at the scale of UK Biobank and beyond (i.e. for millions of variants and up to 500 000 individuals) to be carried out effectively, we introduce *GWAIS-Web*, an ultra-fast, convenient and secure epistasis detection web service with a seamless integration of GPU and FPGA hardware accelerators. *GWAIS-Web* enables genetics researchers to easily perform PLINK’s “gold-standard” logistic regression test plus further implemented methods for GWAS case-control data sets free of charge without requiring their own compute resources. In addition, we improved the stand-alone software *HybridGWAIS* (which acts behind the user interface of *GWAIS-Web*) and provide the source code in publicly available Github projects (see Data availability).

## 2 GWAIS-Web overview

*GWAIS-Web* provides a convenient user interface (UI) as a web service to our epistasis detection software *HybridGWAIS*. In comparison to our previous implementation from Wienbrandt *et al*. (2019), we substantially improved *HybridGWAIS* in terms of speed, efficiency, flexible resource utilization and support for additional second- and third-order epistasis screening methods (such as BOOST (Wan *et al*. 2010) and entropy-based tests) besides PLINK’s second-order logistic regression Wald test, which we have now also implemented for third-order (i.e. SNPxSNPxSNP) interactions. Additional features include, the ability to run multiple interaction tests simultaneously in one run (without increasing runtime) and outputting only a user-selectable number of top association results according to interaction score or *P*value. *GWAIS-Web* now supports runtime prediction before starting the job, on-the-fly filtering of SNP pairs by linkage disequilibrium (LD) for both second- and third-order tests and options for selecting chromosomal regions of interest (e.g. for interaction testing only within known GWAS risk loci or for SNPs within/outside the extended human Major Histocompatibility Complex (xMHC) on chromosome 6). We also distribute the computational workload to several GPU and FPGA accelerators in an automated way, which drastically reduces runtime.

Because genomic data is personal data, we must ensure compliance with privacy-by-design requirements (Pardau and Edwards 2017). Compared to genotype imputation servers (McCarthy *et al*. 2016), where uploading own GWAS data sets is common practice, *GWAIS-Web* implements various additional security measures for the transmission and temporary storage of (sensitive) genetic data, including the exclusive use of encrypted and certified connections (externally certified https), password-protected data access (external downloads via terminal are possible only with a one-time password), optional 2-factor authentication (2FA) (via passkeys, key dongles and/or TOTP authenticator apps) and the protection of all user account data (server hardening). This supports users comply with data privacy laws such as the European General Data Protection Regulation (GDPR).

As GWAIS analyses may run for several hours, user accounts are required to manage job submissions, provide access to result files, and enable email notifications upon job completion. To minimize the collection of personal data, registration requires only a valid email address and a password (with optional two-factor authentication). Job submissions are queued using the workload manager *SLURM* (Jette and Wickberg 2023). The jobs are queued on a first-come, first-serve basis, but to ensure fair usage of our servers and to prevent individual users from monopolizing the queue, we limit the maximum number of queued jobs to three jobs per user, and, based on a runtime estimation, we allow jobs to be queued only if they are expected to finish in less than 24 hours. Further, uploaded data must correspond to a case-control dataset in PLINK’s bed/bim/fam format and should preferably be quality-controlled (e.g. using *BIGwas* (Kässens *et al*. 2021)) to reduce the chance of false positives. The .bim and .fam files must not exceed 100 MB while the file size of the .bed file is automatically checked to be consistent with .bim and .fam before the upload.

We present a list of our newly implemented algorithmic features in comparison to PLINK 1.9 in Table 1, and a summary of the most important technical aspects of the *GWAIS-Web* web service are summarized in Table S1, available as supplementary material at *Bioinformatics Advances* online.

**Table 1:** Comparison of the algorithmic features of *GWAIS-Web* versus PLINK 1.9.

Feature	GWAIS-Web	PLINK 1.9
Accessibility	on- & offline	offline
Hardware accelerator support	✓	×
Acceleration factor	> 2175x	1x
Simultaneous execution of tests	✓	×
Second-order epistasis tests:		
logistic regression	✓	✓
BOOST	✓	✓
log-linear test	✓	×
mutual information	✓	×
information gain	✓	×
Third-order tests:		
logistic regression	✓	×
mutual information	✓	×
information gain	✓	×
LD result filter	✓	×
Proximity exclude range	✓	×
Chromosomal region selection	✓	×
Individual score threshold	✓	✓
Result ordering	✓	×
*n*-best results selection	✓	×
Runtime prediction	✓	×
Floating point precision	double	single

For details on the user interface, the server infrastructure, additional security measures and how we facilitate compliance with the rules of high-standard data privacy laws, please refer to Supplementary Section 1 and Supplementary Figs. 1–5, available as supplementary material at *Bioinformatics Advances* online. Supplementary Section 2, available as supplementary material at *Bioinformatics Advances* online describes the implementation of the *HybridGWAIS* software used behind *GWAIS-Web* including the concept of using contingency tables (Fig. S6, available as supplementary material at *Bioinformatics Advances* online) and all implemented test methods (overview in Table S2, available as supplementary material at *Bioinformatics Advances* online). The hardware acceleration is described in Supplementary Section 3 and Fig. S7, available as supplementary material at *Bioinformatics Advances* online.

We made all new implementations and features as well as the GPU and FPGA hardware resources freely available in *GWAIS-Web*, accessible at https://hybridcomputing.ikmb.uni-kiel.de. The stand-alone software *HybridGWAIS* can be run on any Linux system (with or without GPU and/or FPGA support) (see Data availability).

## 3 Evaluation

For performance benchmarking, we generated simulated GWAS datasets of varying sizes containing up to 2 million SNPs and up to 1 million samples based on the allele frequencies of 3 069 931 variants of chromosome 1 from the Haplotype Reference Consortium (HRC) reference panel r1.1 (McCarthy *et al*. 2016). We further analyzed a real-world quality-controlled GWAS case-control dataset from a COVID-19 study comprising 16 739 samples (4108 cases and 12 631 healthy controls) and 443 401 SNPs. We applied the logistic regression test from the *HybridGWAIS* software with five different accelerator modes directly on our backend system of *GWAIS-Web* and measured the speedup relative to PLINK 1.9 (Chang *et al*. 2015). Details on how we created the benchmark datasets and the benchmark setup are described in Supplementary Section 4.1, available as supplementary material at *Bioinformatics Advances* online. The exact wall-clock runtime measurements can be found in Supplementary Tables 3–9 and Fig. S8, available as supplementary material at *Bioinformatics Advances* online. Note, that *GWAIS-Web* always uses the fastest acceleration mode (acceleration with two FPGAs plus one GPU).

**Figure 1:**
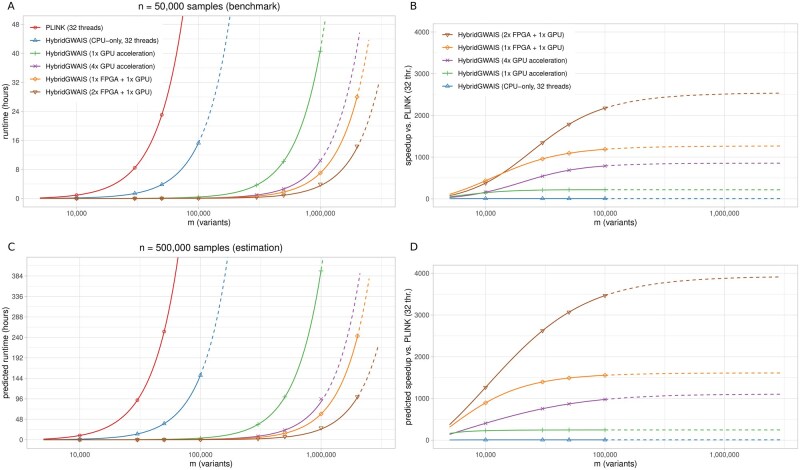
Runtime benchmark and runtime prediction results from five different accelerator modes of *HybridGWAIS*, i.e. (i) CPU-only multi-processing with 32 threads, (ii) acceleration with one GPU, (iii) acceleration with four GPUs, (iv) combined acceleration with one FPGA and one GPU, (v) combined acceleration with two FPGAs and one GPU, compared to the PLINK 1.9 epistasis test with 32 threads, using second-order logistic regression interaction testing as an example. The upper left subfigure **A** shows the runtimes for simulated datasets of a fixed number of *n *= 50 000 samples and a variable number of variants. The upper right subfigure **B** shows the respective speedups of the five modes in *HybridGWAIS* compared to PLINK 1.9 with 32 threads. The lower subfigures **C** and **D** show a simulated benchmark by illustrating the predicted runtimes and speedups for a fixed number of *n *= 500 000 samples (which corresponds to the sample size in UK Biobank) and a variable number of variants. The *x*-axes of all subfigures are in logarithmic scale. Solid lines in the runtime graphs (left subfigures) represent the quadratic fitting of data points. Dotted lines represent the extrapolation of these functions. Solid lines in speedup graphs (right subfigures) represent the relation of PLINK’s fitted runtime function to the fitted functions of each acceleration mode, respectively.

Figure 1A shows the runtimes of *HybridGWAIS* in different accelerator modes compared to PLINK 1.9 with 32 threads for the pairwise logistic regression test for a fixed number of *n *= 50 000 samples and a variable number *m* of variants. For *m *= 100 000 we measured runtimes between 2 minutes and 30 seconds for acceleration with two FPGAs plus one GPU and 15 hours 17 minutes 25 seconds for the CPU-only mode (Supplementary Tables 4–8, available as supplementary material at *Bioinformatics Advances* online). In contrast, the PLINK 1.9 runtime was 3 days 18 hours 31 minutes (Table S3, available as supplementary material at *Bioinformatics Advances* online). As expected, the runtime of *HybridGWAIS* increases approximately quadratically with the number of variants and linearly with the number of samples. For a fixed number of samples and a variable number of variants, we expected a quadratic runtime behavior which was confirmed in our figures.

PLINK 1.9 surprisingly shows a more than linear runtime increase with increasing sample size (see Supplementary Tables 3 and 9, and Fig. S8, available as supplementary material at *Bioinformatics Advances* online for benchmark results for a fixed number of *m *= 50 000 variants and a variable number of samples *n*) and its runtime increased impracticably for a larger sample size than *n *= 50 000 (Table S3, available as supplementary material at *Bioinformatics Advances* online). This observation is underlined by the increase in speedup of *HybridGWAIS* with an increasing number of samples (Fig. 1B). For example, we observe a speedup of about 2175x for *n *= 50 000 samples and *m *= 100 000 variants when using acceleration with two FPGAs plus one GPU (which is the default acceleration mode in *GWAIS-Web*) compared to PLINK 1.9. A *GWAIS-Web* analysis with 50 000 samples and one million variants takes 3 hours, 55 minutes and 49 seconds in this accelerator configuration.

Next, we predicted by linear extrapolation how the runtime benchmark for pairwise logistic regression would behave for a fixed number of *n *= 500 000 samples (which corresponds to the sample size of the UK Biobank (Backman *et al*. 2021)) and a variable number *m* of variants (Fig. 1C). Runtime prediction was necessary because the runtime of PLINK 1.9 already exceeded 2 days with *m *= 10 000 (Table S3, available as supplementary material at *Bioinformatics Advances* online) so that runtime measurements and speedup comparisons for a higher number of variants are practically impossible with PLINK 1.9. For *m *= 100 000 we predicted a PLINK 1.9 runtime exceeding 41 days. In contrast, *HybridGWAIS* would require between 17 minutes and 18 seconds for two FPGAs plus one GPU acceleration and more than 6 days for the CPU-only mode. The predicted speedup is about 3466x for two FPGAs (Fig. 1D), emphasizing the need for algorithmic improvements and runtime acceleration (see Table S10, available as supplementary material at *Bioinformatics Advances* online for all runtime predictions with *n *= 500 000 samples). A large-scale GWAIS involving 500 000 samples (comparable to the size of UK Biobank) and one million genome-wide variants could be completed in approximately 1 day, 3 hours and 55 minutes using two FPGAs and a single GPU. In contrast, PLINK 1.9 running on 32 threads would require more than 10 years to process such a dataset, making it impractical for GWAIS at this scale.

Further, we measured the impact of simultaneously performing multiple second-order epistasis tests on the runtime for *n *= 100 000 samples and *m *= 100 000 variants (Table S11, available as supplementary material at *Bioinformatics Advances* online). The runtime in the accelerator configuration with two FPGAs and one GPU ranges from 285 seconds (using e.g. mutual information tests alone) to 289 seconds (for the combination of all available methods) which means a deviation of only −0.43%–0.9% compared to an analysis using the logistic regression method alone (286 seconds). Similar results are observed for the other accelerator modes, which shows that the simultaneous execution of different test methods in one run has only a negligible influence on the runtime.

Finally, to demonstrate applicability and to verify the correctness of our pairwise association results from *GWAIS-Web*, we evaluated a real-world case-control GWAS dataset from a COVID-19 study (Degenhardt *et al*. 2022). We compared the top 1 000 000 results of our implementation to the results of PLINK 1.9 (after filtering numerically instable results with odds-ratios of zero or infinity) and calculated the Pearson Correlation Coefficient (PCC) and the Mean Relative Error (MRE) of the *χ*^2^ test statistic. The PCC for this example is 0.9996 while the MRE is 0.0014 with a standard deviation of 0.0022, demonstrating a strong concordance of the results with almost no deviation. Regarding the runtime, PLINK required more than 20 days (483 hours and 37 minutes) while *GWAIS-Web* finished after only 33 minutes using two FPGAs plus one GPU, corresponding to a speedup of 867.91x. This demonstrates that also in real-world data scenarios *GWAIS-Web* produces results nearly identical to those of PLINK (with negligible deviations), but substantially faster. Detailed results from the COVID-19 benchmark can be found in Table S12, available as supplementary material at *Bioinformatics Advances* online.

## 4 Conclusion

*GWAIS-Web* is a secure, convenient and freely accessible web service that combines FPGA and GPU acceleration to enable ultra-fast epistasis screening for genome-wide association interaction studies (GWAIS). Researchers benefit from the superior performance enabled by combined FPGA and GPU acceleration without having to purchase and install the necessary hardware themselves.

We offer a variety of different epistasis screening methods (currently to analyze binary traits in case-control datasets) that can be run in parallel without additional runtime overhead. Since both user data (personal data of registered users) and genetic data (personal data of GWAS participants) are temporarily processed and stored as part of our service, we have implemented *GWAIS-Web* in accordance with the requirements of the concept of privacy by design (Pardau and Edwards 2017), to help users comply with data privacy regulations. This includes a high level of data and account security, transparency about data processing, no disclosure of data, no links to external websites, use of strictly functional cookies only and no forms of unwanted data collection. By default, users can submit jobs with a predicted maximum runtime of 24 hours (precomputed client-side before uploading any PLINK format genotype data) which is sufficient for a huge variety of large GWAS datasets with more than 500 000 samples and SNPs. For predicted runtimes of more than 24 hours, we ask interested users to contact us directly so that we can exceptionally allow longer runtimes without blocking the service for other users.

Future work will include adjustment for population structure using covariates (such as principal components from a Principal Component Analysis (PCA)) as well as the investigation of higher-order interactions. Further, we plan to include the implementation of linear regression for quantitative traits and support for processing genotype dosage data from genotype imputation.

## Supplementary Material

vbag172_Supplementary_Data

## Data Availability

The *GWAIS-Web* web service is accessible at https://hybridcomputing.ikmb.uni-kiel.de. It is based on the combination of two separate software projects: (a) *HybridGWAIS* (stand-alone software for epistasis detection with optional GPU support; written in C++ for Linux; accessible under GNU GPL v3.0 license at https://github.com/ikmb/hybridgwais); (b) *HybridGWAIS-FPGA* (source code for the FPGA hardware design to be used in *HybridGWAIS*; written in VHDL; requires the *AMD Vivado Design Suite*  https://www.amd.com/en/developer/resources/vivado.html to compile the FPGA design incl. required IP cores and an *Alpha Data ADM-PCIE-8K5* FPGA accelerator card https://alpha-data.com/product/adm-pcie-8k5/; accessible under GNU GPL v3.0 license at https://github.com/ikmb/hybridgwais-fpga). Small simulated GWAS input files with simulated interaction effects for a quick analysis of testing pairwise analyses or third-order analyses with *GWAIS-Web* or *HybridGWAIS* can be found at https://github.com/ikmb/hybridgwais/tree/main/example/. All simulated GWAS datasets used in our runtime benchmarks (Supplementary 4.1, available as supplementary material at *Bioinformatics Advances* online) are available at https://hybridcomputing.ikmb.uni-kiel.de/downloads.
