# Supplementary material for: GWAIS-Web: a free and secure web service for ultra-fast and large-scale genome-wide association interaction studies
Source: Bioinform Adv. 2026 Jun 17;6(1):vbag172. doi: 10.1093/bioadv/vbag172 (PMC13302774; doi:10.1093/bioadv/vbag172)
Supplement: vbag172_Supplementary_Data [file vbag172_supplementary_data.pdf]

GWAIS-Web: A Free and Secure Web Service for Ultra-Fast and Large-Scale Genome-wide Association Interaction Studies – Supplementary Material

Lars Wienbrandt, Christoph Prieß and David Ellinghaus

June 15, 2026

Contents

1 *GWAIS-Web* **2**
1.1 *GWAIS-Web* user interface and operation . . . 2
1.1.1 Overview . . . 2
1.1.2 Registration and login . . . 2
1.1.3 Job submission . . . 2
1.1.4 Job queuing system . . . 4
1.1.5 Job management and result download . . . 6
1.1.6 Account management . . . 7
1.2 *GWAIS-Web* server infrastructure . . . 8
1.3 Protection of personal data and additional security measures . . . 9

2 The *HybridGWAIS* software and enhancements **11**
2.1 Overview . . . 11
2.2 Contingency tables . . . 12
2.3 Test methods . . . 12
2.3.1 Logistic regression . . . 12
2.3.2 BOOST and log-linear test . . . 14
2.3.3 Entropy-based tests . . . 16
2.3.4 Linkage disequilibrium . . . 17

3 FPGA and GPU acceleration in *HybridGWAIS* **19**
3.1 FPGA-based creation of contingency tables . . . 19
3.2 GPU-based creation of contingency tables . . . 20
3.3 GPU-based computation of statistical tests . . . 20
3.4 Chromosomal region selection . . . 20
3.4.1 Region selection options . . . 20
3.4.2 Processing of regions with CPU-only or GPU-only acceleration . . . 21
3.4.3 Processing of regions with combined FPGA and GPU acceleration . . . 21
3.4.4 Proximity exclude range . . . 21
3.5 Data collection and post processing . . . 22

4 Supplementary benchmark information **23**
4.1 Creation of benchmark datasets and benchmark setup . . . 23
4.2 Supplementary benchmark results . . . 24

1 *GWAIS-Web*

1.1 *GWAIS-Web* user interface and operation

1.1.1 Overview

A GWAIS job submission in *GWAIS-Web* is started by uploading case-control genotype data in PLINK's `.bed/.bim/.fam` format, which can be done via the web browser, a provided URL or via SFTP. The user can choose from different epistatis screening methods (either for 2nd-order (pairwise) or 3rd-order interactions) including PLINK's logistic regression Wald test, BOOST, log-linear test and entropy-based measures such as mutual information and information gain (see **Section 2.3** for available methods). The linkage disequilibrium measure (LD as r^2) can optionally be added to the selected methods for on-the-fly calculation and/or on-the-fly filtering of results, as well as many other runtime options. On-the-fly filtering with LD as well as enabling a *proximity exclude range* can be used to reduce false positive results in the result list. In contrast to classical LD-filtering that removes entire variants from a dataset, on-the-fly pairwise filtering only excludes pairs in LD without affecting other combinations of the involved variants. The *proximity exclude range* excludes pairs from testing that reside within a close genetic distance to each other.

After launching a job, the job's progress can be supervised by the user when logged-in, and the job continues to run after logout. When the run is finished, the service sends a notification email to the user that the results are available for download from the user's job administration section. The download can be started either directly in the browser or via a script in a command line terminal. We present a summary of the most important technical aspects of the *GWAIS-Web* web service in **Supplementary Table 1**. The workflow is explained in the following **Sections 1.1.2-1.1.6** and illustrated with screenshots in **Supplementary Figures 2-5**. The server infrastructure is described in **Section 1.2** and details on our data protection policy and additional security measures are summarized in **Section 1.3**.

1.1.2 Registration and login

GWAIS-Web requires the registration of a personal user account including a valid email address. The user account enables the protection against unauthorized access by third-parties to user data and test results. The email address is used to inform the user about her/his job events (such as a job completion, because a genome-wide screening process may take several hours). We explicitly point out that we do not use the provided email address for purposes other than job notifications and account management and do not collect any usage information or statistics of our service in connection with user accounts.

To complete the registration process, the user gets a verification email from our server to the provided email address. (For sending mails the tool *mSMTP* is used.) The email contains a one-time link that finally validates the email address and activates the user account. It is valid for 7 days, after which the account will automatically be deleted if it was not activated before.

The account protection is implemented either via a simple password or optionally for extra security, the user may register a TOTP authenticator app and/or passkeys for 2-factor authentication (see **Section 1.1.6** below).

After login, the user may submit jobs, manage ongoing or completed jobs, download results, and manage the account. Note, that the web service will logout the user automatically after 30 minutes of inactivity.

In general, we renounce the usage of cookies, but to verify the current login status we need to store a necessary session cookie.

1.1.3 Job submission

New jobs are arranged in a queue to ensure a fair order of execution among users (on a first-come, first-served basis). To run a job within our service, the upload of case-control genotype data in PLINK's `.bed/.bim/.fam` format is required. We suggest to conduct a quality-control (QC) prior to uploading the data (e.g. using the *BIGwas* [1] pipeline) to reduce the chance of false positive results.

Supplementary Table 1: Overview of features implemented in *GWAIS-Web*.

Free and simple account registration:<br>• Only email address and password required<br>• Download of personal account information<br>• Delete account option
Data privacy and security:<br>• Data protection policy on website<br>• Optional 2-factor authentication (2FA) via TOTP (authenticator apps) and/or passkeys (smartphone fingerprint, iCloud KeyChain, USB key dongle, etc.)<br>• Certified encrypted connection<br>• No 3rd party data sharing or data transfer to commercial cloud or other external systems<br>• No advertisements, no tracking, no cookies (a necessary session cookie is stored as long as the user is logged in)
Data integrity check already in web browser from client computer before any data upload.
HybridGWAIS process options for method selection, region selection and result filtering available (see **Table 1** in the main paper for details).
Hardware acceleration (GPU/FPGA).
Job control dashboard:<br>• Live online monitoring of job progress<br>• Background processing (no need to stay logged in)<br>• Notification email for finished jobs<br>• All output files (including result and log files) are made available for download, even when the job is cancelled or fails.
Download via browser or terminal:<br>• Secure download with access protection and encrypted connection (no need for extra encryption of data files)
Message board.

A screenshot of the job submission form is depicted in **Supplementary Figure 1**. Uploading a file triplet is possible in three different ways. The easiest way is to upload via a browser. Files can simply be selected in a file selection dialog or dragged and dropped in the designated box. Alternatively, the web server can actively fetch the files from public URLs (e.g. pointing to a private server) that are provided in the submission form. Or the upload via *Secure File Transfer Protocol (SFTP)* can be chosen, which requires the user to provide the host URL, login credentials and relative paths to the files on that server. Note that we use the login credentials only for the purpose of downloading the files. We never submit them in plain-text, as SFTP is an encrypted connection, and we delete them immediately after the upload to our server is finished.

During the upload process the files are checked for consistency to improve reliability and ensure stable job runs. In particular, in the case of a browser upload, a JavaScript module executed on the client's device checks selected files already before upload. The user gets an immediate feedback about files not matching the required restrictions:

- A file triple in PLINK's bed/bim/fam format must be uploaded with the correct endings `*.bed`, `*.bim` and `*.fam`.
- The maximum size of the `.bim`-file or the `.fam`-file must not exceed 100 MB.
- The `.bim`-file and the `.fam`-file are parsed on the client side to determine the number of variants, and the number of cases and controls beforehand. Thus, these files need to have at least 6 columns. Data that contains no cases or no controls is rejected beforehand. The same applies to files with no data at all.
- The `.bed`-file is checked for consistency, i.e. it must contain PLINK's file signature in the first three bytes and the file size must conform to the number of markers in the `.bim`-file and the number of samples in the `.fam` file.

If the user selects another upload method, the files are checked on our server while uploading and the upload stops immediately if a file is not valid.

The `.bim` file is also used to enable the selection of regions of interest within the provided markers (see **Section 3.4**). Other runtime options include an arbitrary job name, the number of results in the output list, and, certainly, the selection of epistasis testing methods for this job including a linkage disequilibrium (r^2) filter. (Currently, *GWAIS-Web* supports the methods listed in **Supplementary Table 2** in **Section 2.3**.)

When submitting the job, the values in the form are translated by a PHP script in the background to the command line options required for the *HybridGWAIS* software. The relative location of the job folder, that is uniquely created for the input, output and log files, is submitted along with the command-line options to the job queuing system *SLURM* (see **Section 1.1.4**).

The queuing status and progress of the job can be supervised in the *Jobs* section. Once a job has finished, the web service gets notified via a secured notification URL (allowing only connections from the backend system). According to the jobs return state (success or failed), the web service generated download links for the result files and notifies the user per email (see **Section 1.1.5**).

1.1.4 Job queuing system

For job queuing we use the freely available process management software *SLURM* [2], version 24.11. *SLURM* is configured to use the complete backend system exclusively for each *GWAIS-Web* job, i.e. the call to the *HybridGWAIS* launch script is enqueued in the *SLURM* queue and executed on the backend system whenever its resources are available. The parameters applied to the launch script are generated from the user options in the *GWAIS-Web* user interface together with the (relative) location of the user's input data. The script converts the relative location to an absolute path and launches the *HybridGWAIS* executable for the user's input files with added default and user options.

Each *SLURM* job is configured with an epilog command that is executed when the job terminates. It is used to notify the web service about the end of the job processing (either successful or not). This invokes several operations on the frontend, such as changing the job state, notifying the user and preparing the download URLs.

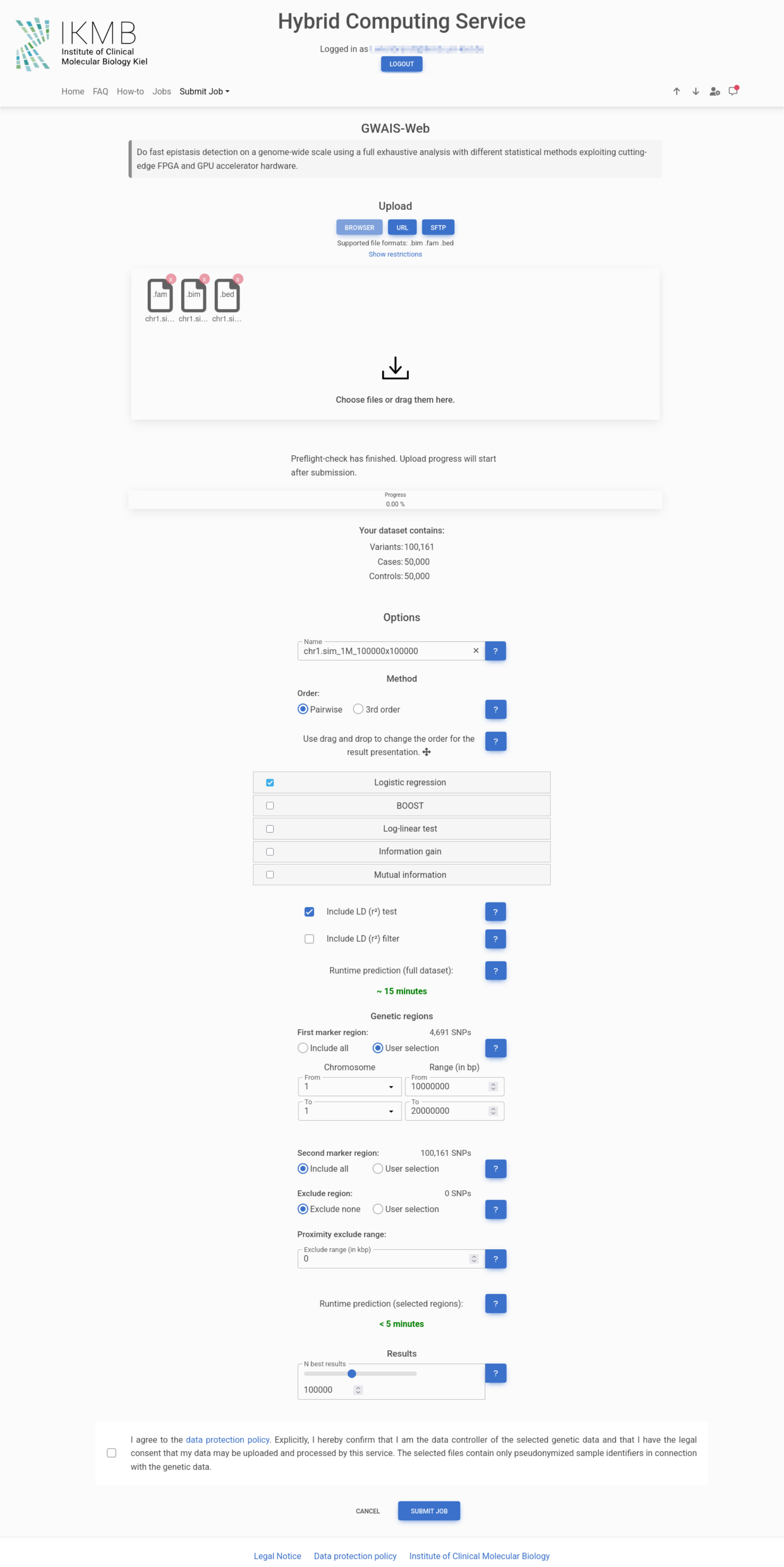

Supplementary Figure 1: Screenshot of *GWAIS-Web*'s job submission page with the available configuration and region selection options.

1.1.5 Job management and result download

After submission, the job is queued in the job queue and can be managed in the "Jobs" section. Each job is classified into four main categories: *queued*, *running*, *terminated* or *retired*, whereby *terminated* can be one of the subcategories *succeeded*, *failed* or *cancelled*. The jobs are listed in chronological order along with a waiting status indicating the position in the job queue. A single user can queue up to three jobs at a time (i.e. no more than three jobs can be queued waiting to be processed by a single user, however, there is no limit to the total number of jobs for a user).

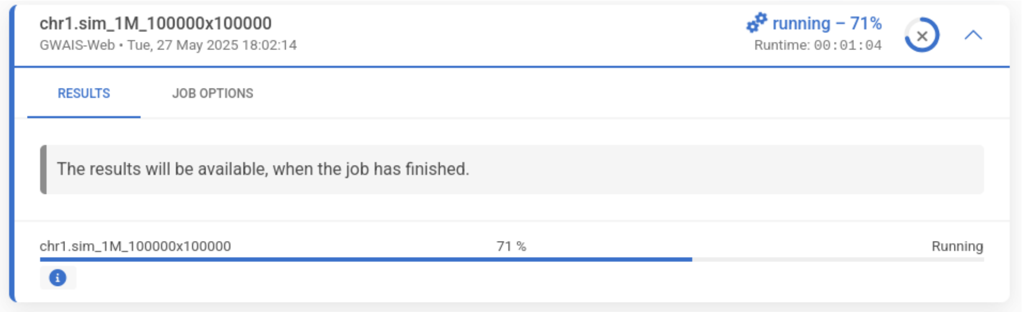

Supplementary Figure 2: Screenshot of an exemplary job progress in *GWAIS-Web*.

Supplementary Figure 2 shows a screenshot from an exemplary job progress. Once a job is actively being processed, the user can monitor the current progress and view information about the job, such as the elapsed runtime, warning and error messages. When a job is regularly completed, it is classified as *succeeded* and the result files can be downloaded. A job can also be cancelled by the user, in which case it is stopped immediately and classified as *cancelled*. If an error occurs, job processing stops and it is classified as *failed*. However, log files of the job execution can still be downloaded even for failed or cancelled jobs. In all cases, users receive a notification email with information on the job's termination status.

For each successfully finished job the web service generates a `.scores`-file that contains the n best results of the analysis according to the first selected test method, whereby n was also selected by the user. Alternatively, the user may have selected a fixed score threshold, in which case the `.scores`-file contains all scores exceeding the threshold but capped by the best 1 million results. The score file is provided as a zipped `.gz`-file. For the detailed explanation of the contents in the `.scores`-file see the methods descriptions in **Section 2.3**. In addition, the *HybridGWAIS* execution log is provided in a `.log`-file.

The result files are registered in our database with a unique random string for each file. The web service then generates a secure download URL for each file over an encrypted *https*-connection based on this random string. We use the *rewrite engine* by the Apache server to decode this URL to redirect a download request to our download engine, and the download engine queries the database for the associated file. **Supplementary Figure 3** shows a screenshot from a finished job with exemplary result files.

The user may download each result file separately from the web browser, but an individual script to download all available files at once from a command line terminal is also provided to the user. For this purpose, the user has to request a one-time password uniquely associated with that job. The files are locked by default and a direct download via the file URL is only possible when the received one-time password is entered via the download script on the command line to authenticate the download. Technically, the password has to be sent as a *POST* command after establishing the encrypted *https* connection to our externally certified web server to download a file, which is done automatically by the provided script. This is advantageous if the result files have to be analyzed or post-processed on a different system than the user's computer. The one-time password expires after a single usage for each file. If it is not used, it expires automatically after 12 hours. This way, separate encryption of the result files is not required because the connection is encrypted and the access to the download link as well as the request to the one-time password is secured by the user's login method (which should preferably be enhanced with 2-factor authentication, see below). This saves the user time by not having to decrypt the files before using.

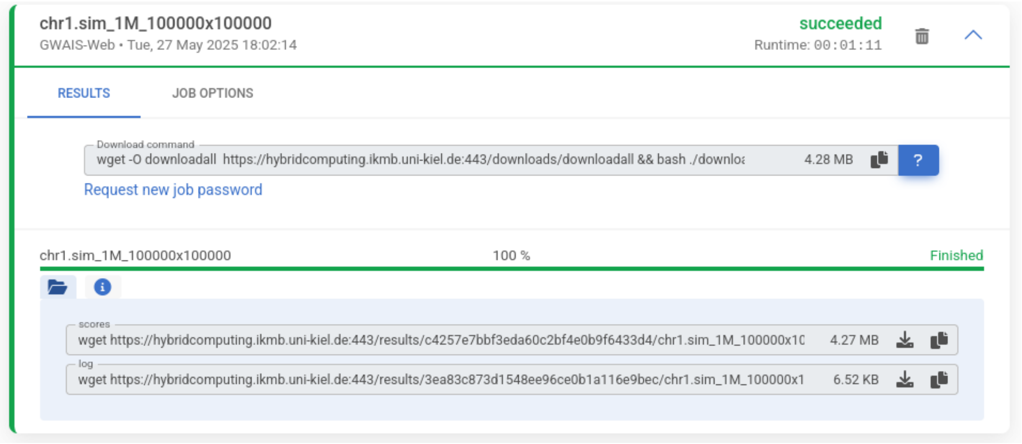

Supplementary Figure 3: Screenshot of a finished job with exemplary result files in *GWAIS-Web*.

Note, that we automatically retire a job after 7 days after the job has terminated. In that case all data related to a job (with the exception of status and log files) is deleted from our server. We keep status and log files to allow users to recall information from their previously run jobs. However, this does not apply to a manual deletion by the user or retired jobs older than a year in which cases everything is deleted completely.

1.1.6 Account management

To set up a personal user account, only a valid email address and a password are required. The password is not stored in plain text, but as a secure SHA-256 hash on our server. Users are able to change their email address and password at any time in the account management section.

GWAIS-Web also provides a password recovery function to set a new password in the case the user has forgotten the login password. The user can click on the link "Forgot your Password?" below the password field and enter the email address and a captcha code displayed as image, which is necessary to prevent abuse by bots. If the captcha code is correct and the email address is registered in our database, a link to reset the password is sent to the user. After clicking the link, a new random password is sent to that email address. The user can now sign in again and change the password.

Optionally, to improve account security, the user can activate 2-factor authentication (2FA) for her/his account based on the *Time-based One-Time Password (TOTP)* [3] and/or *Web Authentication API (Webauthn)* [4] standard. TOTP-based authentication requires an authenticator app (such as *Google Authenticator*) while Webauthn enables usage of hardware authenticators with public and private key-based credentials to perform an SSL handshake between server and the client's authenticator as trusted device. A trusted device may be a USB key dongle, fingerprint reader or facial recognition on a phone or any other applicable device. In detail, an account which is protected by 2FA requires the correct password and the correct authentication of one of the registered 2FA methods to verify the user's identity upon login. Note, that after the registration of a 2FA method, the login is not possible without the second factor, especially if a trusted device was selected, the login is not possible from clients where this device is not available, e.g. if a smartphone's integrated fingerprint reader is used as the only trusted device, the login is not possible from devices other than that smartphone. **Supplementary Figure 4** shows an exemplary registered 2FA method in the account settings.

The user may also delete its account in the account settings, which results in an immediate deletion of all data associated with this account from our server without exception. Specifically, the login credentials are removed from our database together with all uploaded files and data created for this user as a result from using the service.

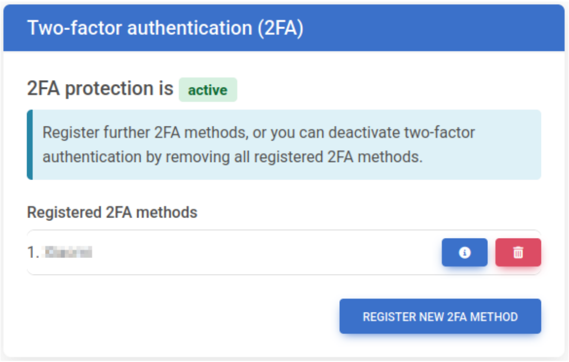

Supplementary Figure 4: Screenshot of exemplary account settings with a registered 2-factor authentication method.

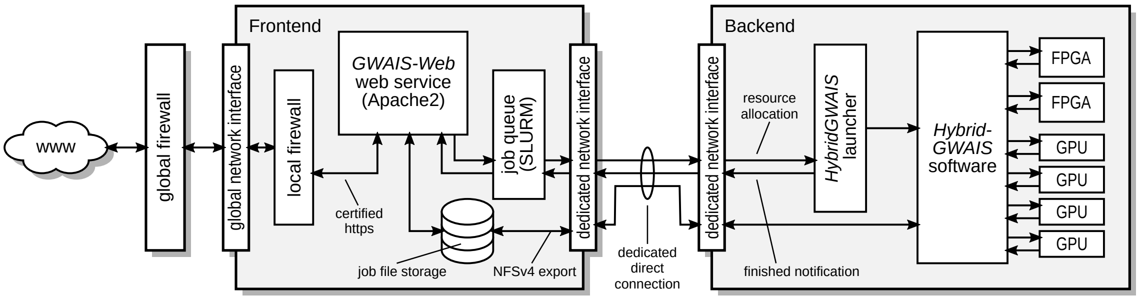

Supplementary Figure 5: The *GWAIS-Web* server architecture consists of two main components: the frontend and backend systems. The web service is hosted by the frontend while jobs are processed on the backend system. The processing order is controlled by the job queuing software *SLURM*. Files are stored on the frontend and accessed via an *NFSv4* network file system.

1.2 *GWAIS-Web* server infrastructure

An overview of the server architecture of *GWAIS-Web* is depicted in **Supplementary Figure 5**. It is divided into two components, referred to as *frontend* and *backend* system, to make our service fast, stable and secure. The frontend hosts the web service including a database with user information, such as login credentials and all data regarding submitted jobs. Uploaded files and result files are also stored on this system. The frontend is equipped with an Intel Xeon Silver 4110 8-core CPU @ 3 GHz and 128 GB RAM. It offers more than 7 TB of redundant storage capacity and is currently running on an Ubuntu 24.04.4 LTS Linux system, which is regularly updated. The web service is mainly written in *PHP* and *JavaScript* and is hosted by an *Apache2 v2.4* [5] server. The database is implemented in *PostgreSQL* [6], version 16.9. For job queuing, we use *SLURM* [2], version 24.11.

With two Intel Xeon Gold 6134 8-core CPUs running at @ 3.2 GHz and 768 GB RAM, the *backend* provides the necessary computing power for the actual processing of the submitted jobs. The operating system is Ubuntu Linux 24.04.4 LTS as well. The backend is further equipped with two FPGA accelerators (Alpha Data ADM-PCIE-8K5 PCIe containing an AMD Kintex UltraScale KU115 FPGA each), and four Nvidia Tesla V100 GPUs. The backend's main task is to run the *HybridGWAIS* tool [7] with FPGA-extension [8] to process the user's epistasis screening jobs. *HybridGWAIS* is written in *C++* using *CUDA* and compiled with *GCC v13.3.0*. The FPGA extension is written in *VHDL* using the *AMD Vivado* [9] developing platform. See **Section 2** for details on *HybridGWAIS*.

To ensure direct communication without potential interception risks and routing problems, the frontend and backend are connected via a direct Ethernet connection and dedicated network devices. Communication between these systems via this connection is limited to two applications: First, the backend offers access to its *SLURM* daemon over this direct connection from the fron-

tend. Second, the frontend only exports the storage file system via *NFSv4* to the backend via this connection and accepts communication to the *SLURM* process. A firewall on the frontend (implemented with the Linux system tool *iptables*) rejects all incoming traffic from the internet except *https* requests. (In particular, *http* requests are also allowed, but are automatically redirected to *https*.) The backend firewall is configured to completely block all incoming traffic from the internet. Only administrative access via *SSH* is exceptionally allowed only from the local network for both systems.

Since the servers are located in the infrastructure of Kiel University, an additional firewall from the university's router ensures that rules are not violated. The server's certificate (required for secure *https* connections) is issued via Kiel University by the external organization *Hellenic Academic and Research Institutions CA* and will be verified by any browser's standard certificate chain.

1.3 Protection of personal data and additional security measures

As genome-wide data is personal data, we must fulfill the requirements of privacy by design [10]. There are many international examples of legislation implementing privacy by design. In the US, this principle has been enshrined in the California Consumer Privacy Act (CCPA), among others, and in 2012 the US Federal Trade Commission (FTC) published a framework of best privacy practices for implementing privacy and data security for businesses that collect and use consumer data [11]. In Europe, privacy by design is explicitly mandated in the European Union's General Data Protection Regulation 2016/679 (GDPR) [12]. How we facilitate compliance with the rules of data privacy laws is explained in detail in our privacy policy on our web website [13]. In brief, we do not use the provided email address for other purposes than job and account management, we do not use the uploaded genetic data for other purposes than the execution of the analysis request, and no one other than the user has access to the data. All data remains locally on our university server located in Kiel, Germany, and all uploaded files and result files (except log and status files) are automatically deleted 7 days after completion of the corresponding job. No data is passed on to third-party services, the website is free of advertising and does not store any cookies on the user's computer (except for a technically necessary session cookie). For reasons of transparency, the user can also download all personal data stored on our server in a single file with a single mouse click. All user-related data is immediately deleted if the user decides to delete the account (which can be done directly by the user in the user administration section).

Our web service features additional security measures to prevent unauthorized access already partially mentioned the explanation of the user interface in Section 1.1 and the server infrastructure in Section 1.2. In summary, we list the most important features here:

- User accounts can optionally be protected with 2-factor authentication which prevents e.g. password fishing attacks. We do not force the use of 2FA as we leave it to the user to decide whether their data needs additional protection.
- The web service is only accessible via externally certified and encrypted *https* connections. Unencrypted *http* connections are automatically redirected to *https*. External certification ensures a direct connection to the server (excluding man-in-the-middle attacks).
- The servers are physically located in and maintained by the University of Kiel, Germany. We do not use cloud services and do not transfer data to other servers than those described here, especially not to servers outside of Germany or the European Union in general (an inevitable exception is the download of data from users outside Germany). We renounce the use of cookies or advertisements, although a necessary session cookie on the client side is essential to keep track of the login status of the user.
- The access to the servers is only possible for users via the web interface. Administrative access is restricted to password-less login via SSH only from the university's Virtual Private Network (VPN), explicitly excluding external connections. This protection is ensured by a global firewall hosted by the university and a local firewall implemented with *iptables*. Further, administrative actions on the servers (e.g. changes of configuration files) are logged.

- The compute server (*backend*) is connected to the server hosting the web service (*frontend*) via a dedicated direct network connection, i.e. no data communication between those two servers can physically be intercepted by other systems. Further, data is shared between these systems via dedicated, secured and encrypted NFSv4.

- Password are never stored or transferred in clear text. Instead, connections are generally encrypted and passwords are stored as a SHA-256 hash in our database.

- Downloads from external client terminals (other than the system the user is logged-in) is possible for convenience, but additionally protected with a random one-time password generated from the user interface (i.e. the user has to authenticate and login prior to being able to request that password). Further, download URLs include a randomly generated hash unique for each file making the access impossible for users other than the owner.

- We further guarantee that data uploaded to our servers will only be used for the purpose of the analysis and the results will solely be made available to the user. All input and result data can be deleted anytime by the users themselves (by simply deleting the job). Further, all job data will automatically be deleted after 7 days from finishing the job (if not already done by the users themselves) by a cleaning daemon running on the server. Please find our *Data protection policy* on our website for details: `https://hybridcomputing.ikmb.uni-kiel.de/webservice/sites/data_protection_policy.php`

2 The *HybridGWAIS* software and enhancements

2.1 Overview

The core idea of *HybridGWAIS* was to accelerate PLINK's [**?** 14] multiplicative logistic regression epistasis detection test by reducing the computational effort by using a contingency table instead of a covariate matrix [15]. By creating the contingency tables on an FPGA accelerator and running the statistical tests on a GPU, we had achieved a speedup of a factor of more than 1000x compared to the original PLINK 1.9 software. Now, we improved the speed even further to achieve a speedup of more than 3000x (in the order of 50,000 variants and 100,000 samples) and made the *HybridGWAIS* software used in *GWAIS-Web* freely available as a stand-alone software at GitHub [7, 8]. To support GWAIS researchers who do not have access access to FPGA or GPU accelerator hardware or do not want to use *GWAIS-Web*, *HybridGWAIS* is implemented that it can optionally be used with GPU accelerators alone or parallelized across multiple threads to be used without any hardware accelerators at all on a desktop PC or computing cluster node with a multicore CPU.

Since case-control datasets can generally have genotype information condensed into contingency tables, many different kinds of statistical epistasis tests can be conducted on them. In our new *HybridGWAIS* software, we therefore re-use the generated contingency tables for all user-selected epistasis tests, and as the contingency table generation takes most of the computational time, the analysis of a dataset can be done by conducting several tests at the same time without significant increase in runtime. Besides PLINK's multiplicative logistic regression test, we implemented BOOST [16], log-linear regression (i.e. BOOST without pre-filtering), and the entropy-based tests mutual information and information gain. Because ideally the variants to be tested should not be in linkage disequilibrium (LD) with each other, an LD-test calculating the r^2-value between two markers can optionally be added as a new feature in addition to the selected methods. The user may also select any r^2-threshold to filter variant pairs in LD on-the-fly (though only the specific pair in high LD will be filtered out; the variants of this pair will still be used for pairings with other variants and thus remain available for the exhaustive search). We also included third-order tests to analyze marker triples instead of only marker pairs. The LD-test and LD-filter then apply to all pairwise combinations in each marker triple. The results of all tests are summarized in an output table showing the x best results according to the test selected first. (The parameter x is freely selectable by the user.) The result table is implemented with the help of a *MinMaxHeap* where the minimum and maximum elements can be accessed in constant time and the insertion of an element requires logarithmic time ($\mathcal{O}(\log x)$).

Furthermore, *HybridGWAIS* now allows for arbitrary selection of chromosomal regions (e.g. known GWAS regions) to conduct only a subset of all possible tests, thus significantly reducing the analysis runtime and the search space. Regions can be selected independently for each pair/triple index, making it is possible to test only markers from a certain chromosome or genetic region (e.g. from the human major histocompatibility complex (MHC)) to another chromosome or genetic region. Each region can be specified as an interval with any start and end position in the input dataset. It is also possible to additionally exclude a region with arbitrary start and end position, either completely or only for the first marker. This allows analyses e.g. for a selected chromosome against the rest of the dataset (excluding the chromosome) or analyses of one specific region against another region (overlapping or not). Users can set a *proximity exclude range* measured in base pairs. If two markers of a pair or triple are within this range, the combination will not be selected for testing. This can be helpful when marker pairs that are close to each other generate a high statistical score but are unlikely to form an epistatic relationship due to their proximity.

With the ability to focus only on specific regions, *HybridGWAIS* is also able to automatically distribute the analysis across multiple FPGAs and GPUs to further increase computational speed. The *GWAIS-Web* web service takes advantage of this by using up to two FPGAs and four GPUs for one analysis, depending on the size of the GWAS dataset.

In the following, the basic concepts of using contingency tables in the *HybridGWAIS* software and details on the implemented test methods are explained (**Sections 2.2 and 2.3**).

2.2 Contingency tables

For any variant pair (A, B) a contingency table represents the number of samples in a dataset that carry a specific genotype information. In particular, an entry n_{ij} represents the number of samples that carry the information $g_A = i$ at variant A and $g_B = j$ at variant B. Thus, a contingency table for pairwise genotypic tests contains 3×3 entries. Since we are focusing on binary traits, we require a contingency table for each state, w.l.o.g. one for the *case* and one for the *control* group, respectively, and denote their entries by n_{ij}^{case} and n_{ij}^{ctrl} (see **Supplementary Figure 6**).

cases $(Y = 1)$		variant A: 0	1	2
variant B	0	n_{00}^{case}	n_{01}^{case}	n_{02}^{case}
	1	n_{10}^{case}	n_{11}^{case}	n_{12}^{case}
	2	n_{20}^{case}	n_{21}^{case}	n_{22}^{case}

controls $(Y = 0)$		variant A: 0	1	2
variant B	0	n_{00}^{ctrl}	n_{01}^{ctrl}	n_{02}^{ctrl}
	1	n_{10}^{ctrl}	n_{11}^{ctrl}	n_{12}^{ctrl}
	2	n_{20}^{ctrl}	n_{21}^{ctrl}	n_{22}^{ctrl}

Supplementary Figure 6: Contingency tables for cases and controls. n_{ij} reflect the number of occurrences for the corresponding genotype combination in a given pair of variants.

2.3 Test methods

Currently, *HybridGWAIS* supports the test methods listed in **Supplementary Table 2**. In addition, the user may add the calculation of linkage disequilibrium (*LD* as r^2) to the selected methods. It is also possible to enable pairwise/triplewise filtering based on LD on-the-fly. All methods are described in the following.

Supplementary Table 2: Currently supported test methods available in *HybridGWAIS* and *GWAIS-Web*.

**2nd-order (pairwise) methods**	**3rd-order methods**
logistic regression	logistic regression
BOOST	
log-linear test	
mutual information	mutual information
information gain	information gain
**optionally appendable to at least one selected method:**	
linkage disequilibrium (r^2)	linkage disequilibrium (all pairwise r^2)

2.3.1 Logistic regression

The logistic regression test implemented in *HybridGWAIS* is taken from PLINK's [**?**] multiplicative logistic regression model, with β_3 indicating the interaction effect:

$$\ln \frac{P(Y = 1|X_A = g_A, X_B = g_B)}{P(Y = 0|X_A = g_A, X_B = g_B)} = \beta_0 + \beta_1 g_A + \beta_2 g_B + \beta_3 g_A g_B \tag{1}$$

Y defines the categorization if a sample is *case* $(Y = 1)$ or *control* $(Y = 0)$. X_A and X_B represent random variables correlated with the observation of genotypes at variants A and B, respectively. The possible outcomes of $X_{A/B}$ are $g_{A/B} \in \{0, 1, 2\}$ representing the observed genotype (0 = homozygous reference, 1 = heterozygous, 2 = homozygous variant).

The model is now fitted using the previously generated contingency tables described in **Section 2.2** and an iterative Newton method. For the first iteration, we start with $\boldsymbol{\beta} = (0, 0, 0, 0)$. The iterations are then processed as follows:

1. For each sample i, compute intermediate variables

$$p_{ij}^{(t)} = \left(1 + e^{-\left(\beta_0^{(t)} + i\beta_1^{(t)} + j\beta_2^{(t)} + ij\beta_3^{(t)}\right)}\right)^{-1} \tag{2}$$

$$p_{ij}^{(t),\mathrm{ctrl}} = p_{ij}^{(t)}, \quad p_{ij}^{(t),\mathrm{case}} = p_{ij}^{(t)} - 1, \quad v_{ij}^{(t)} = p_{ij}^{(t)}\left(1 - p_{ij}^{(t)}\right)\left(n_{ij}^{\mathrm{case}} + n_{ij}^{\mathrm{ctrl}}\right) \tag{3}$$

2. Compute gradient

$$\boldsymbol{\nabla}^{(t)} = \left(\sum_{ij} N_{ij}^{(t)}, \sum_{ij} iN_{ij}^{(t)}, \sum_{ij} jN_{ij}^{(t)}, \sum_{ij} ijN_{ij}^{(t)}\right) \tag{4}$$

   where

$$N_{ij}^{(t)} = \left(n_{ij}^{\mathrm{case}} p_{ij}^{(t),\mathrm{case}} + n_{ij}^{\mathrm{ctrl}} p_{ij}^{(t),\mathrm{ctrl}}\right) \tag{5}$$

3. Compute symmetric Hessian matrix

$$\boldsymbol{H}^{(t)} = \left(h_{pq}^{(t)}\right)_{p,q=0}^{3} = \begin{pmatrix} \sum v_{ij}^{(t)} & & \cdots & \\ \sum iv_{ij}^{(t)} & \sum i^2 v_{ij}^{(t)} & & \vdots \\ \sum jv_{ij}^{(t)} & \sum ijv_{ij}^{(t)} & \sum j^2 v_{ij}^{(t)} & \\ \sum ijv_{ij}^{(t)} & \sum i^2 jv_{ij}^{(t)} & \sum ij^2 v_{ij}^{(t)} & \sum i^2 j^2 v_{ij}^{(t)} \end{pmatrix} \tag{6}$$

   where each sum is evaluated over all indexes i and j

4. Compute $\boldsymbol{\Delta\beta}^{(t)} = \left(\Delta\beta_j^{(t)}\right)_{j=0}^{3}$ by efficiently solving the linear system

$$\boldsymbol{L}^{(t)} \boldsymbol{L}^{(t)T} \boldsymbol{\Delta\beta}^{(t)} = \boldsymbol{\nabla}^{(t)} \tag{7}$$

   using the Cholesky decomposition $\boldsymbol{L}^{(t)} = \left(l_{jk}^{(t)}\right)_{j,k=0}^{3}$ of $\boldsymbol{H}^{(t)}$ with

$$l_{jk}^{(t)} = \begin{cases} 0 & \text{if } k > j \\ \sqrt{h_{jj}^{(t)} - \sum\limits_{s=1}^{j-1} l_{js}^2} & \text{if } k = j \\ \frac{1}{l_{kk}}\left(h_{jk}^{(t)} - \sum\limits_{s=1}^{k-1} l_{js} l_{ks}\right) & \text{if } k < j \end{cases} \tag{8}$$

5. Update model parameters

$$\boldsymbol{\beta}^{(t+1)} \leftarrow \boldsymbol{\beta}^{(t)} - \boldsymbol{\Delta}\beta^{(t)} \tag{9}$$

If $\sum_j \Delta\beta_j^{(t)}$ approaches zero, i.e. there is no more significant change (≤ 0.0001), the process stops with $\boldsymbol{\beta}^{(t+1)}$ as the current result. Otherwise, the next iteration is started with step 1. However, if the change does not converge to zero, the process stops after a fixed number of iterations (currently 16 which was taken over from PLINK).

The result of the logistic regression test in PLINK is composed of three components, namely the test statistic, its approximate p-value and the odds-ratio. The test statistic χ^2 is calculated as a Wald test:

$$\chi^2 = \frac{\beta_3^2}{\varepsilon^2}. \tag{10}$$

ε is the standard error for the $g_A g_B$-term in Eq. 1. It can directly be determined by solving the linear system $\boldsymbol{H}^{(t)}\boldsymbol{e} = (0, 0, 0, 1)$ and defining $\varepsilon^2 = e_3$.

Accordingly, it follows

$$\varepsilon = \left| \frac{1}{l_{33}^{(t)}} \right| . \tag{11}$$

The test statistic is assumed to follow a chi-squared distribution χ_1^2 with one degree of freedom. Accordingly, the p-value can directly be approximated from the respective cumulative distribution function (CDF):

$$\text{Pval}(x) = 1 - \text{CDF}(\chi_1^2(x)) \tag{12}$$

Finally, the odds-ratio is defined as

$$OR = e^{\beta_3}. \tag{13}$$

For a third-order logistic regression test the following multiplicative logistic regression model is fitted analogue to the procedure described above:

$$\ln \frac{P(Y=1|X_A=g_A, X_B=g_B, X_C=g_C)}{P(Y=0|X_A=g_A, X_B=g_B, X_C=g_C)} = \beta_0 + \beta_1 g_A + \beta_2 g_B + \beta_3 g_C + \beta_4 g_A g_B + \beta_5 g_A g_C + \beta_6 g_B g_C + \beta_7 g_A g_B g_C \tag{14}$$

The same definitions as above apply plus X_C and g_C describe the random variable and its outcomes of the third variant C. The reported interaction effect is taken from β_7.

Logistic regression test results: The following results are reported by *HybridGWAIS* for the logistic regression test in the `.scores`-file.

`POS_A`	Genetic position in the format *chr:bp* of variant A.
`POS_B`	Genetic position in the format *chr:bp* of variant B.
`POS_C`	Genetic position in the format *chr:bp* of variant C (optional).
`SNPID_A`	Identifier string of variant A.
`SNPID_B`	Identifier string of variant B.
`SNPID_C`	Identifier string of variant C (optional).
`LOGR_CHISQ`	χ^2-score calculated according to Eq. (10).
`LOGR_OR`	odds-ratio OR calculated according to Eq. (13).
`LOGR_P-VAL`	p-value of the χ^2-score assuming a χ^2-distribution with one degree of freedom, calculated according to Eq. (12).
`LOGR_BETA`	value of β_3 after the last iteration used to calculate the χ^2-score in Eq. (10).
`LOGR_EPS`	the standard error ε after the last iteration according to Eq. (11).

2.3.2 BOOST and log-linear test

In BOOST [16] an interaction is defined as the difference between the log-likelihoods of the saturated model $\hat{L}_S$ and the homogenous model $\hat{L}_H$. As no closed solution for the homogenous model exists, the BOOST method was divided into two parts.

BOOST pre-filter: At first, the *Kirkwood Superposition Approximation (KSA)* of the homogenous model $\hat{L}_{KSA}$ is calculated. Wan et al. showed that the difference of the log-likelihoods is an upper bound to the desired calculation of the interaction effect:

$$\hat{L}_S - \hat{L}_H \leq \hat{L}_S - \hat{L}_{KSA} \tag{15}$$

This correlation is used as a pre-filter, so the log-linear test (i.e. fitting of the homogeneous model) is done only when the pre-filter exceeds a certain threshold τ, which is fixed to $\tau = 15$ according to [16]:

$$\hat{L}_S - \hat{L}_{KSA} \overset{!}{\geq} \tau \tag{16}$$

The approximated interaction effect is then calculated by:

$$\hat{L}_S - \hat{L}_{KSA} = n \sum_{ijY} \left[\hat{\pi}_{ijY} \log \frac{\hat{\pi}_{ijY}}{\hat{p}^K_{ijY}} \right] \tag{17}$$

where $\hat{\pi}_{ijY}$ is the joint distribution of the saturated model, which can be replaced by the observed relative probability of a certain genotype combination ij for a certain trait Y (n is the number of samples):

$$\hat{\pi}_{ijY} = \frac{n^Y_{ij}}{n} \tag{18}$$

Further, $\hat{p}^K_{ijY}$ is the KSA of the distribution obtained under the homogeneous association model and can be computed as:

$$\hat{p}^K_{ijk} = \frac{1}{\eta} \frac{\pi_{ij\cdot}\pi_{i\cdot k}\pi_{\cdot jk}}{\pi_{i\cdot\cdot}\pi_{\cdot j\cdot}\pi_{\cdot\cdot k}} \tag{19}$$

whereby

$$\eta = \sum_{ijY} \frac{\pi_{ij\cdot}\pi_{i\cdot Y}\pi_{\cdot jY}}{\pi_{i\cdot\cdot}\pi_{\cdot j\cdot}\pi_{\cdot\cdot Y}}. \tag{20}$$

(Note, we use the dot notation to indicate a sum over a subscript, e.g. $\pi_{ij\cdot} = \sum_Y \pi_{ijY}$ and $\pi_{\cdot\cdot Y} = \sum_{ij} \pi_{ijY}$.)

Log-linear test: If the approximated interaction effect using the KSA pre-filter exceeds the predefined threshold of $\tau = 15$, BOOST computes the log-linear test to obtain a better approximation to the desired interaction effect using the homogeneous model $\hat{L}_S - \hat{L}_H$. In *HybridGWAIS*, the user may choose the log-linear test as a separate method to directly calculate the interaction effect without the BOOST pre-filter based on the KSA.

The log-linear test is computed using *Iterative Proportional Fitting* of the homogenous association model using a maximum of 34 iterations until the error is below $\varepsilon^{(t)} \leq 0.001$ (according to the original implementation from [16]). The process starts at iteration $t = 1$ and is initialized with $\mu^{(0)}_{ijY} = 1$.

1. Compute intermediate μ'_{ijY} for all ijY:

$$\mu'^{(t)}_{ijY} = \mu^{(t-1)}_{ijY} \frac{n^{\cdot}_{ij}}{\mu^{(t-1)}_{ij\cdot}} \tag{21}$$

2. Compute intermediate μ''_{ijY} for all ijY:

$$\mu''^{(t)}_{ijY} = \mu'^{(t)}_{ijY} \frac{n^Y_{i\cdot}}{\mu'^{(t)}_{i\cdot Y}} \tag{22}$$

3. Compute μ_{ijY} for all ijY:

$$\mu^{(t)}_{ijY} = \mu''^{(t)}_{ijY} \frac{n^Y_{\cdot j}}{\mu''^{(t)}_{\cdot jY}} \tag{23}$$

4. Compute error ε:

$$\varepsilon^{(t)} = \sum_{ijY} \left| \mu^{(t)}_{ijY} - \mu^{(t-1)}_{ijY} \right| \tag{24}$$

The interaction score is finally calculated as:

$$\hat{L}_S - \hat{L}_H \approx 2 \sum_{ijY} n^Y_{ij} \log \frac{n^Y_{ij}}{\mu^{(t)}_{ijY}} \tag{25}$$

HybridGWAIS reports the interaction score with the last error $\varepsilon^{(t)}$ and an approximated p-value for the interaction score under the assumption of a χ^2-distribution with four degrees of freedom.

Note that BOOST and the log-linear test are not yet implemented as third-order methods in *HybridGWAIS*.

BOOST test results: The following results are reported by *HybridGWAIS* for the BOOST test in the `.scores`-file.

`POS_A`	Genetic position in the format *chr:bp* of variant A.
`POS_B`	Genetic position in the format *chr:bp* of variant B.
`SNPID_A`	Identifier string of variant A.
`SNPID_B`	Identifier string of variant B.
`BOOST_CHISQ`	χ^2-score according to Eq. (25) of the final log-linear interaction test applied after KSA-filtering.
`BOOST_ERR`	standard error ε according to Eq. (24) from the last iteration of the log-linear test applied after KSA-filtering.
`BOOST_P-VAL`	p-value calculated from the χ^2-score test result from Eq. (25) assuming a χ^2-distribution with four degrees of freedom.

Log-linear test results: The following results are reported by *HybridGWAIS* for the log-linear test in the `.scores`-file.

`POS_A`	Genetic position in the format *chr:bp* of variant A.
`POS_B`	Genetic position in the format *chr:bp* of variant B.
`SNPID_A`	Identifier string of variant A.
`SNPID_B`	Identifier string of variant B.
`LL_CHISQ`	χ^2-score according to Eq. (25) of the final log-linear interaction test.
`LL_ERR`	standard error ε according to Eq. (24) from the last iteration of the log-linear test.
`LL_P-VAL`	p-value calculated from the χ^2-score test result from Eq. (25) assuming a χ^2-distribution with four degrees of freedom.

2.3.3 Entropy-based tests

Entropy-based tests in *HybridGWAIS* include *mutual information* and *information gain* (which is also known as *interaction information*) [17, 18]. By defining the observations of the genotypes in a marker pair or triple and the corresponding phenotypes as random variables, one can easily calculate their *entropy* H, which forms the basis of the methods described below. For a random variable X and its possible outcomes $\{x_i\}$ the entropy is defined as

$$H(X) = -\sum_i p(x_i) \log p(x_i). \tag{26}$$

Mutual information: The first method computes the *mutual information (MI)* between the observed genotypes and the corresponding phenotype. Let X_1 be the random variable for observations of genotypes at the first marker in a pair, and X_2 for the second marker. For third-order tests, let X_3 be the random variable for the third marker. In general, let Y be the random variable for the phenotype, i.e. either case or control. Mutual information describes the overlap of the combined entropies for the genotypes and the entropy for the phenotype, i.e. for pairs

$$I(X_1, X_2; Y) = H(X_1, X_2) + H(Y) - H(X_1, X_2, Y) \tag{27}$$

and for triples

$$I(X_1, X_2, X_3; Y) = H(X_1, X_2, X_3) + H(Y) - H(X_1, X_2, X_3, Y) \tag{28}$$

respectively.

Information gain: The second method computes the *information gain (IG)* from the observed genotypes to the corresponding phenotype. It is based on the mutual information of the single random variables to the phenotype and the combined mutual information as calculated above in Eqs. 27 and 28. *HybridGWAIS* calculates IG according to the definition of Jakulin et al. [17, 18], i.e. for pairs

$$I(X_1;X_2;Y) = I(X_1,X_2;Y) - I(X_1;Y) - I(X_2;Y) \tag{29}$$

and for triples

$$\begin{aligned} I(X_1;X_2;X_3;Y) = I(X_1,X_2,X_3;Y) - I(X_1,X_2;Y) - I(X_1,X_3;Y) - I(X_2,X_3;Y) \\ + I(X_1;Y) + I(X_2;Y) + I(X_3;Y) \end{aligned} \tag{30}$$

respectively. Note, that information gain may be negative.

Mutual information test results: The following results are reported by *HybridGWAIS* for the mutual information test in the `.scores`-file.

`POS_A`	Genetic position in the format *chr:bp* of variant A.
`POS_B`	Genetic position in the format *chr:bp* of variant B.
`POS_C`	Genetic position in the format *chr:bp* of variant C (optional).
`SNPID_A`	Identifier string of variant A.
`SNPID_B`	Identifier string of variant B.
`SNPID_C`	Identifier string of variant C (optional).
`MI`	Mutual information $I(X_1,X_2;Y)$ as in Eq. (27) for 2nd-order tests or $I(X_1,X_2,X_3;Y)$ as in Eq. 28 for 3rd-order tests.

Information gain test results: The following results are reported by *HybridGWAIS* for the information gain test in the `.scores`-file.

`POS_A`	Genetic position in the format *chr:bp* of variant A.
`POS_B`	Genetic position in the format *chr:bp* of variant B.
`POS_C`	Genetic position in the format *chr:bp* of variant C (optional).
`SNPID_A`	Identifier string of variant A.
`SNPID_B`	Identifier string of variant B.
`SNPID_C`	Identifier string of variant C (optional).
`IG`	Information gain $I(X_1;X_2;Y)$ as in Eq. (29) for 2nd-order tests or $I(X_1;X_2;X_3;Y)$ as in Eq. 30 for 3rd-order tests.

2.3.4 Linkage disequilibrium

Linkage disequilibrium is usually measured as an r^2-score and is a measure of similarity between two variants. It is defined as

$$r^2 = \frac{D^2}{p_A(1-p_A)p_B(1-p_B)} \quad \text{with} \quad D = p_{AB} - p_A p_B. \tag{31}$$

D is the distance between the observed allele frequency p_{AB} at loci A and B and the expected allele frequency $p_A p_B$ assuming statistical independence. Thus, r^2 is a normalized measure for D which can be used for comparison of different variant pairs. The allele frequencies p_A and p_B can directly be determined as

$$p_A = \frac{2n_{00} + 2n_{10} + 2n_{20} + n_{01} + n_{11} + n_{21}}{2n} \tag{32}$$

and

$$p_B = \frac{2n_{00} + 2n_{01} + 2n_{02} + n_{10} + n_{11} + n_{12}}{2n}, \tag{33}$$

respectively, where $n_{ij} = n_{ij}^{\text{case}} + n_{ij}^{\text{ctrl}}$ for all i, j. Unfortunately, the determination of the allele frequency p_{AB} from genotypic data is not trivial. This is due to the unknown phase when two heterozygous genotypes face each other in a variant pair. Basically, it can be defined as

$$p_{AB} = \frac{2n_{00} + n_{01} + n_{10} + x}{2n} \tag{34}$$

with x meeting $x \leq n_{11}$. x has to satisfy the following equation whose solution is omitted here for simplicity:

$$(f_{00} + x)(f_{11} + x)(n_{11} - x) = (f_{01} + n_{11} - x)(f_{10} + n_{11} - x)x \tag{35}$$

where f_{ij} is the number of allele combinations ij we know for sure, e.g. $f_{00} = 2n_{00} + n_{01} + n_{10}$ and $f_{11} = 2n_{22} + n_{21} + n_{12}$.

Note that in general, there exist more than one solution for this equation. Thus, *HybridGWAIS* calculates and reports the smallest and the largest of the (at maximum three) possible solutions for r^2. If an LD-filter is applied in addition to the test calculation, only pairs with the largest solution for r^2 being below the filter threshold are reported.

For third-order tests the r^2-score is computed for all three pairwise combinations in a variant triple. In contrast to the pairwise method only the largest solution of each of the pairwise calculations is reported. If an LD-filter is applied to third-order methods, triples only pass the filter if all pairwise r^2-scores are below the filter threshold.

Additional results for linkage disequilibrium (2nd-order): The following result columns are added to the `.scores`-file of any 2nd-order (pairwise) interaction test in *HybridGWAIS* when enabling the linkage disequilibrium measure.

- `R2_H` largest (highest) r^2 of possibly three solutions satisfying Eq. (31).
- `R2_L` smallest (lowest) r^2 of possibly three solutions satisfying Eq. (31).

Additional results for linkage disequilibrium (3rd-order): The following result columns are added to the `.scores`-file of any 3rd-order interaction test in *HybridGWAIS* when enabling the linkage disequilibrium measure.

- `R2_AB_H` largest (highest) r^2 of possibly three solutions satisfying Eq. (31) for the pair of variants A and B.
- `R2_AC_H` largest (highest) r^2 of possibly three solutions satisfying Eq. (31) for the pair of variants A and C.
- `R2_BC_H` largest (highest) r^2 of possibly three solutions satisfying Eq. (31) for the pair of variants B and C.

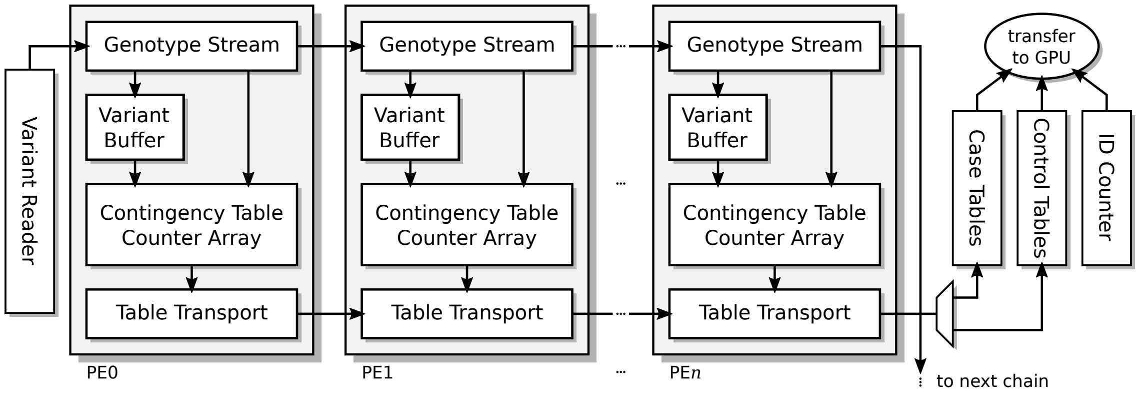

Supplementary Figure 7: Chain of processing elements (PEs) in the FPGA design for creating contingency tables. Our design for the *AMD Kintex KU115* FPGA contains 480 PEs which allows the creation of 480 contingency tables in parallel.

3 FPGA and GPU acceleration in *HybridGWAIS*

3.1 FPGA-based creation of contingency tables

The acceleration method of combining FPGAs and GPUs is divided into two main parts. Firstly, the FPGA accelerator creates the contingency tables for each marker pair or triple, while, secondly, the GPU accelerator processes each contingency table by applying the selected statistical tests. For a complete exhaustive analysis of all marker pairs (or triples) we refer to the method description in [15] and [19]. We shortly summarize the method for the creation of pairwise contingency tables here. The modifications required to handle arbitrary region selections are described below in **Section 3.4**.

The FPGA design targets the *AMD Kintex UltraScale KU115* FPGAs with 16 GB of attached DRAM (distributed over two DRAM modules) on our *Alpha Data ADM-PCIE-8K5* accelerator cards. The design sustains a pipeline consisting of a chain of 480 process elements (PEs) divided into two subchains with 240 PEs each. After a short initialization phase, the chain produces 480 contingency tables concurrently while the genotype data of one marker is streamed through the pipeline at a speed of 266 MHz and 8 genotypes per clock cycle. This sums up to a peak performance of about 20.4 million contingency table pairs per second for an exemplary dataset containing about 50,000 samples.

In detail, each PE in a chain is organized into a memory buffer to store the genotypes of all samples at a single marker position, implemented in local block RAM (BRAM), and a counter array for the contingency table entries implemented in registers (see **Supplementary Figure 7**). The PE is able to receive a stream of genotype data from a previous PE and to process it while also delivering it to the next PE. For each PE the local genotype buffer is filled with the first incoming genotype data for one complete marker. In particular, the data for the first marker is only stored in the local memory and not forwarded to the next PE. Subsequent variants are then forwarded and simultaneously used to form genotype pairs with the data stored in the local memory. Dependent on the current pair, the corresponding counter of the contingency table is incremented. By streaming eight genotypes at once for each marker pair, i.e. incrementing the corresponding counters of eight genotype combinations at the same time, we could achieve a high throughput of genotype data in each PE.

The genotype data for the analysis is stored in the first half of the attached DRAM. Streaming is organized by sending the genotypes of the case samples at the current variant position first, followed by the genotypes of the control samples. Thus, the contingency tables for cases and controls are alternately generated using the same logic resources in the PE.

The contingency tables are retrieved from the PEs again via the PE chain. After all genotypes from either the cases or the controls were streamed, the table is provided to the next PE in one clock cycle. Each PE hands over incoming tables to the next PE first before it sends its own table. All tables are collected at the end of the chain in a separate buffer (implemented in the second

half of the FPGA-attached DRAM) before they are transmitted to the GPU via the host.

Thus, the collection of tables requires as many clock cycles as there are PEs in the chain implying that the minimum number of either cases or controls for the most efficient utilization of the chain is exactly eight times the number of PEs in the chain. In order to prevent delays resulting from collecting the contingency tables, we divided the complete chain into subchains with continuous genotype streaming but separate table collection units.

3.2 GPU-based creation of contingency tables

In the case the FPGA acceleration is disabled or not available the contingency tables can be created directly on the GPU. The host loads the genotype database in the GPU local memory beforehand and provides a continuous flow of buffers to the GPU that contain index pairs indicating which marker pairings are to be analyzed next. For each buffer a number of CUDA threads is launched, each thread processing one pair.

The CUDA thread firstly creates the contingency table by reading the required genotype data from local GPU memory and then continues the calculation of the test statistic in the same way as with an attached FPGA accelerator (see **Section 3.3**). There is no need to store the contingency table anywhere other than in the local thread registers. Thus, we only create warp divergence while reading the individual marker data for each thread, but due to the packed variant-wise data format with a 2-bit encoding per genotype, each thread only requires a few kilobytes which can be processed almost immediately. Furthermore, in most cases, the marker pairs in the current warp share the first marker and the second markers in the pairs are in consecutive order. Together with the word-aligned genotype data, this is an optimal scenario for fast access to the local GPU memory.

3.3 GPU-based computation of statistical tests

In the hybrid solution with combined FPGA and GPU accelerators, the buffers from the FPGA containing contingency tables are transferred to local GPU memory. We use a default transmission buffer size of 256 MB which may hold up to 6.7 million table pairs. In a GPU-only acceleration scenario, the transmission buffers contain only index pairs for which the GPU needs to create the contingency tables from genotype data in the local GPU memory itself (as described above in **Section 3.2**).

The computation process follows a simple parallelization scheme over CUDA threads. By setting the block size to the maximum supported block size and the grid size to evenly distribute the contingency tables over the blocks, each thread processes exactly one contingency table pair, and only one kernel call per buffer is required. Besides the distribution of the contingency table data and the writeback of the results no calls to local GPU memory are required from a GPU thread in the hybrid scenario (in the GPU-only scenario each thread needs to recall the required genotype data from local memory).

The test methods are implemented as described in **Section 2.3** using double precision floating point formats where applicable. (In contrast, PLINK uses only the single precision floating point format in its computations.) The resulting test scores are written into a result buffer. We provide one result buffer for each table transmission buffer, which is transferred to the host as soon as processing the table buffer has finished.

3.4 Chromosomal region selection

3.4.1 Region selection options

In *HybridGWAIS* the user is able to select arbitrary regions to reduce the analysis space by choosing intervals in the input dataset for each variant marker separately. The space usually opens up to $\binom{m}{2} = m(m-1)/2$ pairwise combinations (m is the number of variants) for exhaustive pairwise tests, and $\binom{m}{3} = m(m-1)(m-2)/6$ triple combinations for exhaustive third-order tests. For pairwise tests, users are allowed to concentrate on certain regions by selecting an interval subset for the first marker and another interval subset for the second marker. A third interval may be specified for the third marker when doing a third-order test.

Additionally, users may define an additional interval for a region that they wish to exclude from the analysis. This *exclude region* may be applied either to the complete dataset or only to the interval selection for the first marker. The latter option may be useful if a user wants to test the whole dataset excluding a certain region against the whole data but including that region.

According to the user selections the software simply determines overlapping and non-overlapping regions from the selected intervals. It then determines which tests have to be evaluated and which not. The implementation differs for the selected type of computation whether or not FPGA-acceleration is used (see the following **Sections 3.4.2** and **3.4.3**).

3.4.2 Processing of regions with CPU-only or GPU-only acceleration

If FPGA-acceleration is disabled and the computation is evaluated only on the CPU cores or using GPU-acceleration, the loops that circulate through the number of all tests are programmed to simply leave out regions that are not selected by the user. For CPU-only processing this means that only function calls to variant pairs/triples of interest are performed leaving out unselected combinations. For GPU-acceleration, the host creates lists of pairs/triples to be tested on the GPU anyway, which are then provided to the GPU via the transmission buffers. With enabled region selections the host simply adds only pairs/triples of interest to this list.

3.4.3 Processing of regions with combined FPGA and GPU acceleration

As the FPGA pipeline is designed to concurrently generate contingency tables from a continuous flow of variant data, region selections have to be handled differently when compared to CPU-only or GPU-accelerated processing. The original pipeline designed in [15] was not able to handle region selections. After transmitting the complete dataset to the FPGA-attached local DRAM the pipeline controller starts streaming the complete data through the pipeline. In the first cycle, this generates the contingency tables for the first 480 variants (corresponding to the number of PEs in the pipeline) against all variants in the dataset. In the second cycle, the controller streams the data again, but leaves out the first 480 variants. This effectively generates the contingency tables for the next 480 variants against the remainder. This process continues until the controller cannot start another cycle due to having to leave out all available variants.

In order to handle region selections we modified the pipeline controller in two ways. First, the pipeline cycles can be stopped if the variant to begin a new cycle exceeds a defined index. Second, the controller can "jump" to a certain variant index after the initialization phase of a pipeline cycle. With this modifications the pipeline is able to handle not only regions that completely overlap, but also regions that partially overlap in the beginning or do not overlap at all. As the host system determines these areas of overlap and non-overlap in advance, the FPGA-accelerator can now handle any arbitrary region selections in several runs. Due to the pipeline nature these modifications imply that some contingency tables may be created that are not required for the selected analysis, especially in the initialization phase and in the last pipeline cycle. The tables are still handed over to the GPU-accelerator that computes the selected test statistics, but the results are filtered afterwards by the host. This behaviour is still faster than filtering the unneeded tables beforehand. However, this overhead in computation may have an impact in the runtime, especially if inefficient regions are chosen (e.g. selecting only a single variant in a dataset to be tested against the rest), but in the general case, the impact will be negligible and in most cases the analysis speed will still be faster in comparison to a GPU-only acceleration. Anyway, when using *HybridGWAIS* in our web service *GWAIS-Web*, the runtime prediction computes both expected runtimes (with and without using FPGA-acceleration) and eventually starts the analysis with the smaller predicted runtime.

3.4.4 Proximity exclude range

In addition to the chromosomal region selection abilities presented above, the user may also define a *proximity exclude range*. This range is measured in base pairs and defines an area around every marker where tests should be omitted if the second (or third) marker resides within that range. For example, a proximity exclude range of 50 kbp omits all tests where the two markers in a pair (or any two markers in a triple) are located in proximity closer than 50,000 base pairs. Similar

to the region selection options, the implementation differs for the selected acceleration type. For CPU-only or GPU-accelerated processing the distance is checked before running the test, which effectively skips unwanted tests beforehand. In contrast for FPGA-accelerated runs, the tests are evaluated first and the distance of the pairs is checked afterwards excluding unwanted test results before adding them to the result list.

3.5 Data collection and post processing

Multiple threads on the host system perform the collection and post-processing of results. Results are sorted by the result score of the first user selected test method. For this purpose, a *min-max heap* data structure with a user definable size limit is used. In alternative to the size limit, the user may define a significance threshold which prevents results with a score below this limit not to be inserted into the result heap in the first place. If no additional size limit is set by the user, a default maximum of 1 million results is defined as the size limit of the heap.

Each thread keeps its own instance of a min-max heap to avoid lock conditions. After processing all transmission buffers, the results are merged into a single instance. The final results are written into a tab-delimited table file containing the marker IDs, indices and all available scores. For a detailed explanation of the presented method-dependent results in the results file see **Section 2.3**.

4 Supplementary benchmark information

4.1 Creation of benchmark datasets and benchmark setup

For runtime benchmarking, we used simulated datasets of different sizes with a varying number of markers and samples, but with an equal distribution of cases and controls. First, we generated a large dataset of 1 million simulated individual samples based on the allele frequencies of 3,069,931 variants of chromosome 1 from the *Haplotype Reference Consortium (HRC)* reference panel *HRC r1.1*, freely available at `ftp://ngs.sanger.ac.uk/production/hrc/HRC.r1-1/HRC.r1-1.GRCh37.wgs.mac5.sites.vcf.gz`. For each sample we randomly generated the alleles for each variant according to the allele frequency presented in this file. We converted the resulting VCF file to PLINK's bed/bim/fam format and randomly sampled subsets with different sizes (a different number of variants and samples) from this file using PLINK 1.9. The resulting files were then used for our benchmarks and are available for download at `https://hybridcomputing.ikmb.uni-kiel.de/downloads`.

Second, we benchmarked a quality-controlled GWAS case-control dataset from a COVID-19 study comprising 16,739 samples with 443,401 SNPs [20]. In particular, the dataset contains 4,108 COVID-19 cases and 12,631 healthy controls from five different countries (Austria, Germany, Italy, Norway and Spain). Quality-control was conducted using the BIGwas pipeline [1].

Note, that the most computationally intensive part is the creation of the contingency tables, which is independent of the content of the table. Thus, the total runtime essentially depends only on the size of the dataset, not on its contents or the type of statistical test. (In particular, the content of the tables have a minimally negligible impact on the runtime, since in fitting a logistic regression model, for example, the number of Newton iterations depends on the quality of the result, but is internally capped by a number of 15 iterations anyway.)

Consequently, we initially applied the benchmark process only with the logistic regression method to different combinations of sample and marker sizes. We tested the *HybridGWAIS* software with different accelerator methods on our backend system, i.e. (i) using only multi-processing with 32 threads on our CPUs alone (runtime parameter `-t 32`), (ii) using one GPU accelerator (`--gpu 0`), (iii) using four GPU accelerators (`--gpu 0 1 2 3`), (iv) using a combination of one FPGA and one GPU accelerator (`--gpu 0 --fpga 0`), and (v) using two FPGA accelerators and one GPU accelerator in combination (`--gpu 0 --fpga 0 1`). Note that the web service *GWAIS-Web* uses only the fastest configuration (v) for regular operations. We also conducted a PLINK analysis to measure the speedup of *HybridGWAIS* over PLINK 1.9 [14]. (In the newer PLINK 2.0 software, epistasis testing is not yet implemented.)

```
$ plink1.9 --bfile <infile> --threads 32 --epistasis --out <outfile>
```

To illustrate that the combination of different test methods has a negligible impact on the runtime, we ran several tests with the same dataset (100,000 samples with 100,000 variants), but different combinations of test methods. We ran this benchmark with the same accelerator configurations (ii)-(v) as above. Here, we omitted the CPU-only configuration (i) because the expected runtime was too high and we did not expect different results in terms of runtime deviation compared to the configurations with accelerator(s) used. We measured all runtimes with the standard GNU time command.

We measured the accuracy of our web service in comparison to PLINK by calculating the Pearson Correlation Coefficient (PCC) and the Mean Relative Error (MRE) inlcuding its standard deviation (σ) of the χ^2 test statistic of the R = 1,000,000 top results (after filtering numerically instable results with odds-ratios of zero or infinity).

The PCC is calculated as follows:

$$\mathrm{PCC} = \frac{\sum_{i=0}^{R-1}\left(\chi^2_{i,\mathrm{PLINK}} - \overline{\chi^2_{\mathrm{PLINK}}}\right)\left(\chi^2_i - \overline{\chi^2}\right)}{\sqrt{\sum_{i=0}^{R-1}\left(\chi^2_{i,\mathrm{PLINK}} - \overline{\chi^2_{\mathrm{PLINK}}}\right)^2}\sqrt{\sum_{i=0}^{R-1}\left(\chi^2_i - \overline{\chi^2}\right)^2}} \tag{36}$$

The MRE and σ are calculated as follows:

$$\text{MRE} = \frac{1}{R}\sum_{i=0}^{R-1} \text{RE}_i \tag{37}$$

$$\text{with } \text{RE}_i = \frac{\left|\chi^2_{i,\text{PLINK}} - \chi^2_i\right|}{\left|\chi^2_{i,\text{PLINK}}\right|} \tag{38}$$

$$\text{and } \sigma = \sqrt{\frac{1}{R}\sum_{i=0}^{R-1} \left(\text{RE}_i - \text{MRE}\right)^2} \tag{39}$$

4.2 Supplementary benchmark results

Supplementary Table 3: Wall-clock runtimes in seconds of PLINK's logistic regression epistasis test for simulated datasets with a different number of variants (m) and a different number of samples (n) using 32 computing threads on our benchmark system. We conducted benchmarks for $n = 10,000$ and $n = 50,000$ samples. For higher n we conducted benchmarks only if the linear runtime prediction did not exceeded 48 hours.

	n = 10,000	50,000	100,000	500,000	1,000,000
m = 10,000	290.34	3,315.08	13,898.12	192,001.73	-
30,000	2,605.55	30,428.37	127,807.41	-	-
50,000	8,776.58	83,003.27	261,549.30	-	-
100,000	35,222.08	325,835.10	-	-	-

Supplementary Table 4: Wall-clock runtimes in seconds of *HybridGWAIS* logistic regression test for simulated datasets with a different number of variants (m) and a different number of samples (n) using CPU-only computation with 32 computing threads on our benchmark system. We conducted benchmarks for $n = 10,000$ and $n = 50,000$ samples. For higher n we conducted benchmarks only if the linear runtime prediction did not exceeded 24 hours.

	n = 10,000	50,000	100,000	500,000	1,000,000
m = 10,000	119.57	558.84	1,106.14	5,488.78	10,956.37
30,000	1,041.82	4,970.56	9,872.64	49,213.27	-
50,000	2,869.07	13,802.26	27,410.12	-	-
100,000	11,527.64	55,044.75	-	-	-

Supplementary Table 5: Wall-clock runtimes in seconds of *HybridGWAIS* logistic regression test for simulated datasets with a different number of variants (m) and a different number of samples (n) using GPU acceleration with a single Nvidia Tesla V100 GPU on our benchmark system. We conducted benchmarks for $n = 10,000$ and $n = 50,000$ samples. For higher n we conducted benchmarks only if the linear runtime prediction did not exceeded 24 hours.

	n = 10,000	50,000	100,000	500,000	1,000,000
m = 10,000	9.64	22.54	39.07	171.79	333.26
30,000	36.34	145.06	283.74	1,397.63	2,738.06
50,000	88.83	383.27	756.06	3,765.36	7,380.50
100,000	335.81	1,499.39	2,977.47	14,880.21	29,145.10
300,000	2,955.37	13,329.59	26,521.93	-	-
500,000	8,152.81	36,689.09	73,125.32	-	-
1,000,000	32,499.53	146,168.41	-	-	-

Supplementary Table 6: Wall-clock runtimes in seconds of *HybridGWAIS* logistic regression test for simulated datasets with a different number of variants (m) and a different number of samples (n) using GPU acceleration with four Nvidia Tesla V100 GPUs on our benchmark system. We conducted benchmarks for $n = 10,000$ and $n = 50,000$ samples. For higher n we conducted benchmarks only if the linear runtime prediction did not exceeded 24 hours.

	n = 10,000	50,000	100,000	500,000	1,000,000
m = 10,000	19.38	23.72	30.17	82.19	144.97
30,000	30.07	56.43	95.86	406.62	783.53
50,000	50.15	119.69	218.23	1,013.85	1,974.53
100,000	143.67	409.99	787.36	3,851.66	7,541.66
300,000	1,138.34	3,445.53	6,739.85	-	-
500,000	3,102.81	9,449.59	18,365.00	-	-
1,000,000	12,230.55	37,484.18	73,122.59	-	-

Supplementary Table 7: Wall-clock runtimes in seconds of *HybridGWAIS* logistic regression test for simulated datasets with a different number of variants (m) and a different number of samples (n) using combined FPGA and GPU acceleration with an Alpha Data ADM-PCIE-8K5 PCIe FPGA accelerator board (containing an AMD Kintex UltraScale KU115 FPGA) and an Nvidia Tesla V100 GPU on our benchmark system.

	n = 1,000	5,000	10,000	50,000	100,000
m = 10,000	8.84	7.98	7.45	9.65	14.11
30,000	16.37	14.91	15.42	32.96	60.02
50,000	32.74	30.54	30.11	77.39	146.11
100,000	110.83	101.99	100.77	275.48	536.02
300,000	774.58	846.60	861.49	2,319.71	4,565.70
500,000	2,468.55	2,340.93	2,389.54	6,368.25	12,591.84
1,000,000	9,425.43	8,597.85	9,194.76	25,308.44	50,108.28
2,000,000	37,078.51	33,638.87	34,288.09	100,783.12	199,877.05

Supplementary Table 8: Wall-clock runtimes in seconds of *HybridGWAIS* logistic regression test for simulated datasets with a different number of variants (m) and a different number of samples (n) using combined FPGA and GPU acceleration with two Alpha Data ADM-PCIE-8K5 PCIe FPGA accelerator boards (containing an AMD Kintex UltraScale KU115 FPGA each) and an Nvidia Tesla V100 GPU on our benchmark system.

	n = 1,000	5,000	10,000	50,000	100,000
m = 10,000	7.99	7.79	7.86	9.51	12.16
30,000	15.42	14.84	15.41	22.44	38.91
50,000	34.65	31.75	31.79	45.60	84.80
100,000	88.58	104.79	101.96	150.04	288.01
300,000	725.87	805.68	863.05	1,188.80	2,344.75
500,000	2,151.84	1,871.66	2,294.49	3,227.95	7,074.00
1,000,000	6,957.15	7,602.57	7,575.77	14,148.78	25,734.47
2,000,000	31,138.10	37,226.14	35,574.14	51,941.97	101,012.59

Supplementary Table 9: Wall-clock runtimes in seconds of *HybridGWAIS* logistic regression test for simulated datasets with a fixed number of variants ($m = 50,000$) and a different number of samples (n) using different benchmark configurations. (Supporting data for **Supplementary Figure 8**.)

	n = 1,000	5,000	10,000	50,000	100,000
PLINK (32 thr.)	473.26	3,719.40	8,776.58	83,003.27	261,549.30
CPU-only (32 thr.)	400.78	1,504.99	2,869.07	13,802.26	27,410.12
1xGPU	38.61	57.38	88.83	383.27	756.06
4xGPU	47.78	49.66	50.15	119.69	218.23
1xFPGA + 1xGPU	32.74	30.54	30.11	77.39	146.11
2xFPGA + 1xGPU	34.65	31.75	31.79	45.60	84.80

Supplementary Table 10: Estimated wall-clock runtimes in seconds of *HybridGWAIS* logistic regression test for simulated datasets with a fixed number of samples ($n = 500,000$) and a different number of variants (m) using different benchmark configurations. (Supporting data for **Figure 1(c)** in the main paper.) The runtimes were predicted using a linear function fitted from the values for corresponding m from previous tables.

	PLINK (32 thr.)	CPU-only (32 thr.)	1xGPU	4xGPU	1xFPGA +1xGPU	2xFPGA +1xGPU
m = 10,000	36,466	5,485	171	82	37	30
30,000	334,712	49,212	1,380	404	242	133
50,000	913,036	136,544	3,715	1,005	618	287
100,000	3,584,186	544,612	14,666	3,818	2,302	1,038
300,000	-	-	129,855	29,915	19,927	8,435
500,000	-	-	361,903	81,608	54,254	26,384
1,000,000	-	-	1,424,943	343,813	218,218	100,491
2,000,000	-	-	-	-	875,464	362,534

Supplementary Table 11: Wall-clock runtimes in seconds of *HybridGWAIS* for multiple testing methods and combinations of multiple testing methods. All runs were executed on the same simulated dataset with m=100,000 variants and n=100,000 samples using different acceleration methods (GPU-only with an Nvidia Tesla V100 GPU and combined FPGA and GPU acceleration with one or two Alpha Data ADM-PCIE-8K5 PCIe FPGA accelerator boards respectively (containing an AMD Kintex UltraScale KU115 FPGA each) and the Tesla V100 GPU) on our benchmark system. The deviation is measured in percent (%) from the runtime of the logistic regression test.

Method	GPU accel.		1x FPGA + GPU accel.		2x FPGA + GPU accel.	
	runtime	dev.	runtime	dev.	runtime	dev.
Logistic Regression (LogReg)	2,965.62	0.00%	532.32	0.00%	286.30	0.00%
BOOST	2,960.42	-0.18%	531.64	-0.13%	285.83	-0.16%
Linkage Disequilibrium (LD)	2,951.15	-0.49%	531.33	-0.19%	285.46	-0.29%
Mutual Information (MI)	2,948.55	-0.58%	531.03	-0.24%	285.08	-0.43%
Information Gain (IG)	2,950.01	-0.53%	530.81	-0.28%	285.23	-0.37%
LogReg + LD	2,974.76	0.31%	533.07	0.14%	287.44	0.40%
LogReg + BOOST	2,984.44	0.63%	532.98	0.12%	287.45	0.40%
LogReg + BOOST + LD	2,994.69	0.98%	533.73	0.26%	287.87	0.55%
LogReg + BOOST + LG + MI + IG	3,008.22	1.44%	534.76	0.46%	288.89	0.90%

Supplementary Table 12: Wall-clock runtimes in seconds and speedup of the logistic regression test in *HybridGWAIS* using different benchmark configurations in comparison to PLINK for the real-world case-control GWAS dataset comprising 16,739 samples (4,108 COVID-19 cases and 12,631 healthy controls) and 443,401 SNPs. The test scores from the *HybridGWAIS* runs are identical. For comparison to PLINK we added the Pearson Correlation Coefficient (PCC), Mean Relative Error (MRE) plus standard deviation (σ) based on the top 1,000,000 results at the bottom.

	Runtime (s)	Speedup
PLINK (32 thr.)	1,741,026	1.00
CPU-only (32 thr.)	184,330	9.45
1xGPU	6,123	284.34
4xGPU	2,668	652.56
1xFPGA + 1xGPU	1,929	902.55
2xFPGA + 1xGPU	2,006	867.91
PCC	0.9996	
MRE	0.0014	
σ	0.0022	

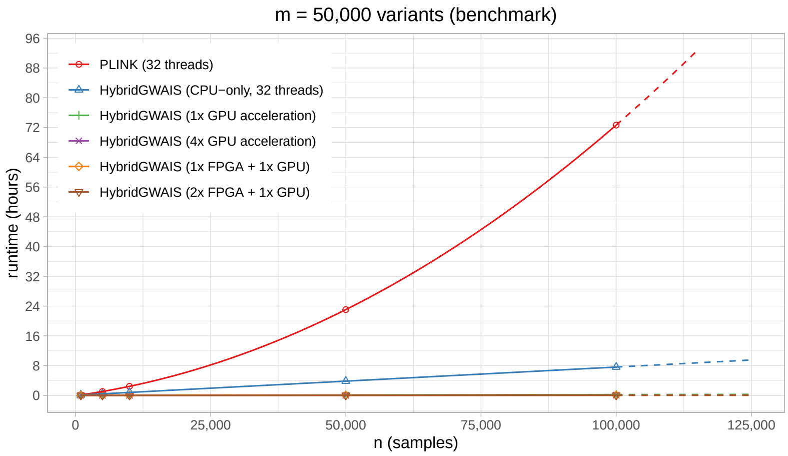

(a)

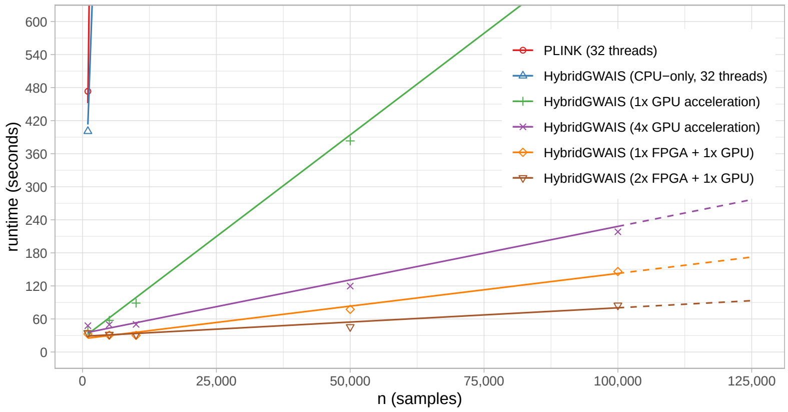

(b)

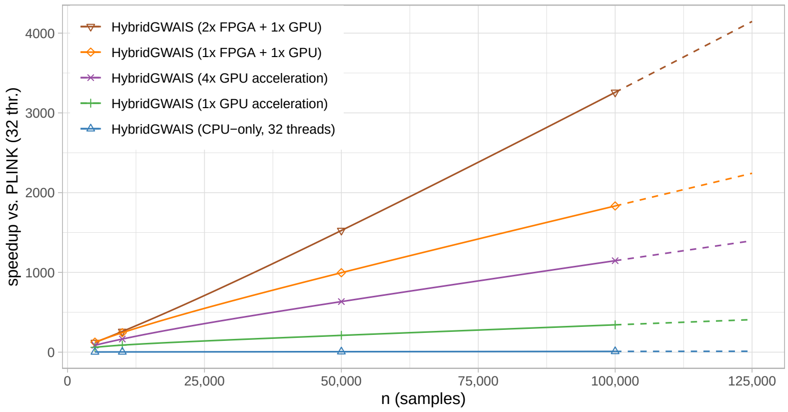

(c)

Supplementary Figure 8: Benchmark results from *HybridGWAIS* compared to the PLINK epistasis test for simulated datasets, using logistic regression as an example. The top figure **(a)** shows the runtimes of a fixed number of $m = 50,000$ variants and a variable number of samples of the epistasis test in PLINK 1.9 running with 32 threads and five different acceleration modes in *HybridGWAIS*: (i) CPU-only multi-processing with 32 threads, (ii) acceleration with one GPU, (iii) acceleration with four GPUs, (iv) combined acceleration with one FPGA and one GPU, (v) combined acceleration with two FPGAs and one GPU. The second figure **(b)** shows a scaled version with a magnified y-axis to highlight the differences between the fastest four acceleration modes. As a consequence, most PLINK and CPU-only data points are not in the visible region anymore. The bottom figure **(c)** illustrates the corresponding speedups versus PLINK 1.9 (using 32 threads). Solid lines indicate the linearly fitted interpolation functions between data points, dotted lines indicate an extrapolation.

References

[1] Kässens, J. C., Wienbrandt, L., and Ellinghaus, D. (June, 2021) BIGwas: Single-command quality control and association testing for multi-cohort and biobank-scale GWAS/PheWAS data. *Gigascience,* **10**(6), giab047.

[2] Jette, M. A. and Wickberg, T. (2023) Architecture of the Slurm Workload Manager. In Klusáček, D., Corbalán, J., and Rodrigo, G. P., (eds.), *Job Scheduling Strategies for Parallel Processing*, Cham: Springer Nature Switzerland pp. 3–23.

[3] M'Raihi, D., Machani, S., Pei, M., and Rydell, J., TOTP: Time-Based One-Time Password Algorithm. Request for Comments 6238, Internet Engineering Task Force (IETF) (May, 2011) RFC 6238.

[4] A simple PHP WebAuthn (FIDO2) server library. `https://github.com/lbuchs/WebAuthn`.

[5] Apache HTTP Server Project. `https://httpd.apache.org`.

[6] PostgreSQL: The World's Most Advanced Open Source Relational Database. `https://www.postgresql.org`.

[7] Wienbrandt, L. HybridGWAIS Software. (2025) `https://github.com/ikmb/hybridgwais`. Accessed 23 December 2025.

[8] Wienbrandt, L. HybridGWAIS FPGA Design Sources. (2025) `https://github.com/ikmb/hybridgwais-fpga`. Accessed 23 December 2025.

[9] AMD AMD Vivado Developer Hub. (2025) `https://www.amd.com/en/developer/resources/vivado.html`.

[10] Pardau, S. L. and Edwards, B. (2017) The FTC, the Unfairness Doctrine, and Privacy by Design: New Legal Frontiers in Cybersecurity. *J. Bus. & Tech. L.,* **12**, 227–276.

[11] Federal Trade Commission Protecting Consumer Privacy in an Era of Rapid Change. (2012) https://www.ftc.gov/sites/default/files/documents/reports/federal-trade-commission-report-protecting- consumer-privacy-era-rapid-change-recommendations/120326privacyreport.pdf. Accessed 08 May 2025.

[12] EU General Data Protection Regulation (GDPR) (2016) Regulation (EU) 2016/679 of the European Parliament and of the Council of 27 April 2016 on the protection of natural persons with regard to the processing of personal data and on the free movement of such data, and repealing Directive 95/46/EC (General Data Protection Regulation). *OJ L 119/1,* pp. 1–88.

[13] Institute of Clinical Molecular Biology Hybrid Computing Service Data Protection Policy. (2025) `https://hybridcomputing.ikmb.uni-kiel.de/webservice/sites/data_protection_policy.php`.

[14] Chang, C. C., Chow, C. C., Tellier, L. C., Vattikuti, S., Purcell, S. M., and Lee, J. J. (December, 2015) Second-generation PLINK: rising to the challenge of larger and richer datasets. *Gigascience,* **4**, 1–16.

[15] Wienbrandt, L., Kässens, J. C., Hübenthal, M., and Ellinghaus, D. (2019) 1000x Faster than PLINK: Combined FPGA and GPU Accelerators for Logistic Regression-based Detection of Epistasis. *Journal of Computational Science,* **30**, 183–193.

[16] Wan, X., Yang, C., Yang, Q., et al. (2010) BOOST: A Fast Approach to Detecting Gene-Gene Interactions in Genome-wide Case-Control Studies. *Am. J. Hum. Genet.,* **87**(3), 325–340.

[17] Jakulin, A. and Bratko, I. (2003) Analyzing Attribute Dependencies. *Knowledge Discovery in Databases: PKDD 2003,* pp. 229–240.

[18] Jakulin, A. and Bratko, I. Quantifying and Visualizing Attribute Interactions: An Approach Based on Entropy. (2004).

[19] Wienbrandt, L., Kässens, J. C., et al. (2017) Fast Genome-Wide Third-order SNP Interaction Tests with Information Gain on a Low-cost Heterogeneous Parallel FPGA-GPU Computing Architecture. *Proc. Computer Science,* **108**, 596–605.

[20] Degenhardt, F., Ellinghaus, D., et al. (November, 2022) Detailed Stratified GWAS Analysis for Severe COVID-19 in Four European Populations. *Human Molecular Genetics,* **31**(23), 3945–3966.
